# Fumed Silica-Derived CoO_x_@SiO_2_ Composites for Catalytic Reduction of 2-Nitrophenol

**DOI:** 10.3390/molecules31132282

**Published:** 2026-06-30

**Authors:** Amina Zharkenova, Aigerim Galyamova, Vassilis J. Inglezakis, Andrey Y. Khalimon

**Affiliations:** 1 Department of Chemistry, School of Sciences and Humanities, Nazarbayev University, 53 Kabanbay Batyr Avenue, Astana 010000, Kazakhstan; amina.zharkenova@nu.edu.kz (A.Z.); aigerim.galyamova@nu.edu.kz (A.G.); 2 Department of Chemical and Process Engineering, University of Strathclyde, Glasgow G1 1XJ, UK; vasileios.inglezakis@strath.ac.uk; 3 Department of Chemical and Materials Engineering, School of Engineering and Digital Sciences, Nazarbayev University, 53 Kabanbay Batyr Avenue, Astana 010000, Kazakhstan

**Keywords:** 2-nitrophenol, reduction, cobalt oxide, emerging pollutants, silica-supported metal oxides

## Abstract

Nitrophenols, despite their wide synthetic utility in the specialty chemical industry, are recognized as toxic and can pose a serious environmental hazard. A popular strategy for the purification of wastewater containing nitrophenols is their catalytic reduction to the corresponding aminophenols in the presence of supported transition metal catalysts. However, the practical application of such systems is hindered by tedious catalyst manufacturing protocols and strong dependence on the catalyst/support microstructure. Herein, a series of CoO_x_@SiO_2_ composites was prepared by a direct reduction of aqueous solutions of Co(II) salts (Co(OAc)_2_, CoCl_2_, and CoF_2_) with fumed silica having a triethoxysilane-modified surface. The prepared composites, despite the observed low cobalt content (0.1–0.2 wt%), proved highly effective in reducing 2-nitrophenol to 2-aminophenol, demonstrating a cost-effective, readily available non-precious-metal-based system for the remediation of nitrophenols from contaminated water.

## 1. Introduction

Nitrophenols are widely used in the manufacture of various chemical products, including dyes, pigments, pesticides, fungicides, and other specialty chemicals [1]. Due to their extensive applications in the chemical industry and their recognized toxicity, nitrophenols can pose a serious environmental hazard when released into wastewater and soil [2]. Thus, the U.S. Environmental Protection Agency (U.S. EPA) has listed nitrophenols among the top-rated pollutants to humans and living organisms [3]. While traditional wastewater treatment methods exhibit relatively low efficacy in removing highly soluble and stable nitrophenols [4], their catalytic reduction to the corresponding aminophenols has emerged as a promising approach [5]. Among various catalytic systems for the reduction of nitrophenols, supported metal catalysts have received particular attention due to their simplicity of preparation, ease of separation from reaction mixtures, and reusability [5,6]. Silica-based supports have emerged as one of the most versatile and widely used materials, owing to their high surface area, thermal stability, chemical inertness, the ease of surface functionalization, and tunable structure [5,6,7,8]. Silica-supported metal catalysts for the reduction of nitrophenols include transition metals such as palladium [9,10,11,12], platinum [9,11,13,14,15], gold [9,11,16,17,18,19,20], nickel [21,22,23,24,25,26], cobalt [6,27,28,29], or copper [30,31,32,33,34] impregnated onto mesoporous or amorphous silica frameworks. The choice of support and the deposition method strongly influence particle size, dispersion of metal species, metal–support interactions, and, hence, the accessibility of catalyst active sites, collectively determining the catalytic performance of the system [35]. Overall, despite significant recent developments in silica-supported metal catalysts for the reduction of nitrophenols, most such systems rely on precious metals (Ru, Pd, Pt, Au, etc.), and catalytic trials are largely limited to 4-nitrophenol (4-NPh) [6,9,10,11,12,13,14,15,16,17,18,19,20]. Indeed, 4-NPh is widely used in the manufacture of pharmaceuticals (such as acetaminophen), fungicides, insecticides, and dyes, and its catalytic reduction to 4-aminophenol (4-APh) is an important industrial transformation [5,36]. Similarly, 3-aminophenol (3-APh) and 2-aminophenol (2-APh) serve as valuable precursors for synthetic dyes and heterocyclic compounds with wide-ranging applications [37]; however, compared to 4-NPh, the reduction of 3-NPh and 2-NPh to the corresponding aminophenols has been less studied [11,38,39,40,41]. In particular, the ortho-isomer is considered less reactive in catalytic reduction due to steric hindrance and intramolecular hydrogen bonding between the adjacent hydroxyl and nitro groups, leading to lower reaction rates and higher activation barriers [38,41].

A popular approach to reduce nitrophenols is their treatment with NaBH_4_ as the reducing agent, which generates reactive metal hydride species on the catalyst surface, thereby promoting hydrogenation of the nitro group [5]. Such transformations have been reported for many noble metal catalysts, including Ru, Pd, Pt, Ag, and Au [5,6,9,10,11,12,13,14,15,16,17,18,19,20]. In recent years, significant research efforts have been focused on improving the sustainability of catalysts for nitrophenol reduction by replacing noble metals with earth-abundant metals and employing cost-effective synthetic methods for catalyst preparation [6,21,22,23,24,25,26,27,28,29,30,31,32,33,34,42]. However, examples of such systems remain scarce, and their practical applications are still hindered [43]. Herein, we report the facile synthesis of cobalt oxide–silica composite catalysts (CoO_x_@SiO_2_) and their use for the efficient reduction of 2-NPh to 2-APh in water. The developed materials are readily accessible from inexpensive cobalt(II) salts (Co(OAc)_2_, CoCl_2_, and CoF_2_) and fumed silica and demonstrate high performance in the reduction of 2-NPh, with reaction rates comparable to those of precious metal surrogates [11].

## 2. Results and Discussion

### 2.1. Preparation and Characterization of Co–O@SiO_2_ Composites

**Figure 1:**
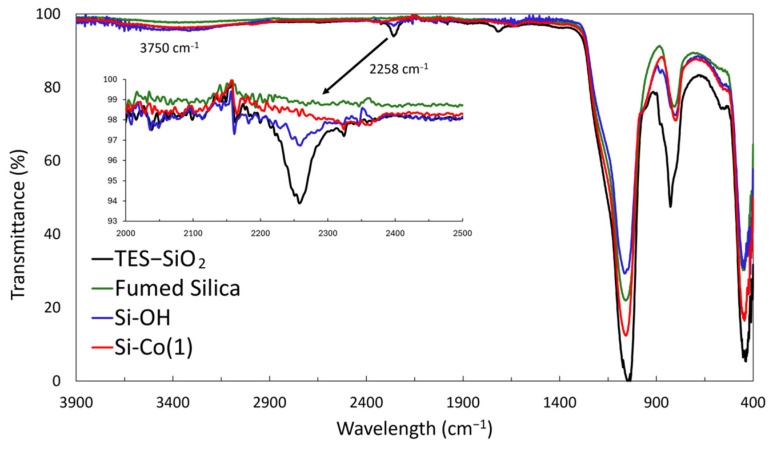
FTIR spectra of fumed silica (green), Si-OH-modified silica (blue), TES–SiO_2_ (black), and Si-Co(1) composite (red).

The strategy for preparing CoO_x_@SiO_2_ composite catalysts was based on a previously developed method for synthesizing noble metal nanoparticles (NPs) on fumed silica [11]. Thus, the commercial fumed silica (395 ± 25 m^2^/g) was first treated with NaOH to increase the Si-OH content (confirmed by Fourier transform infrared spectroscopy (FTIR), showing increased silanol stretching at *υ* 3750 cm^−1^ [44,45,46]), followed by the silica surface modification with triethoxysilane (HSi(OEt)_3_, TES) in glacial acetic acid to generate the Si-H surface groups. The presence of the Si-H functional groups on the silica surface was confirmed by FTIR, which showed the disappearance of O–H stretching vibrations of free surface silanol groups at *υ* 3750 cm^−1^ and revealed a characteristic Si-H stretch at *υ* 2258 cm^−1^ [44,45,46] (Figure 1). The concentration of surface Si-H groups was determined by iodometric titration [45,47], which revealed that 1.1 mmol of ≡Si-H groups per gram of silica were generated.

Given the previously reported application of TES–SiO_2_ as a reductant in the preparation of noble metal NPs/silica composites [11], the prepared TES–SiO_2_ was treated with aqueous solutions of Co(II) salts, Co(OAc)_2_ (OAc = acetate), CoCl_2_, and CoF_2_. These cobalt precursors were selected based on their commercial availability in the region and their likely reactivity with the surface Si-H groups of TES–SiO_2_ due to the affinity of silicon for oxygen and halogens. FTIR analysis of the resulting materials revealed the disappearance of the Si-H stretch (see Figure 1), suggesting the in situ reduction of Co(II) to Co(0), analogous to that of previously tested noble metals (Ru, Pd, Pt, Ag, and Au) [11,46]. The obtained materials were subsequently washed with deionized (DI) water and dried for 18 h at 100 °C under aerobic conditions, which, given the well-known surface instability of Co(0) particles to oxidation [48], afforded the cobalt oxide composites Si-Co(1) (derived from Co(OAc)_2_), Si-Co(2) (derived from CoCl_2_), and Si-Co(3) (derived from CoF_2_).

The content and the oxidation state of the cobalt species in composites Si-Co(1), Si-Co(2), and Si-Co(3) were determined using inductively coupled plasma mass spectrometry (ICP–MS), inductively coupled plasma optical emission spectroscopy (ICP-OES), and X-ray photoelectron spectroscopy (XPS), respectively. Based on ICP-MS and/or ICP-OES, the cobalt content in all prepared composite materials ranged from 1.7·10^−2^ mmol·g^−1^ to 3.4·10^−2^ mmol·g^−1^, with the highest cobalt loading of (2.9 ± 0.5)·10^−2^ mmol·g^−1^ ((1.7 ± 0.3)·10^−1^ wt%) for Si-Co(1), derived from Co(OAc)_2_ (Table 1, entry 1). This was followed by the Si-Co(2) composite formed with CoCl_2_, which showed (2.2 ± 0.2)·10^−2^ mmol·g^−1^ ((1.3 ± 0.1)·10^−1^ wt%) of cobalt (Table 1, entry 2). The material derived from CoF_2_ exhibited the lowest cobalt content, at (2.0 ± 0.3)·10^−2^ mmol·g^−1^ ((1.1 ± 0.2)·10^−1^ wt%; Table 1, entry 3). SEM-EDS (scanning electron microscopy–energy dispersive X-ray spectroscopy) analysis of all three composites showed a similar cobalt content of about 0.1 wt% (see Figures S13–S15 in the Supplementary Materials). Characterization of the Si-Co(1) composite by powder X-ray diffraction analysis (XRD) was also attempted but revealed unresolved spectral bands due to the low Co content (Figure S11). Furthermore, transmission electron microscopy (TEM) of Si-Co(1) showed no agglomeration of cobalt nanoparticles, suggesting dispersion of cobalt species on the silica surface (see Figure S20).

**Figure 2:**
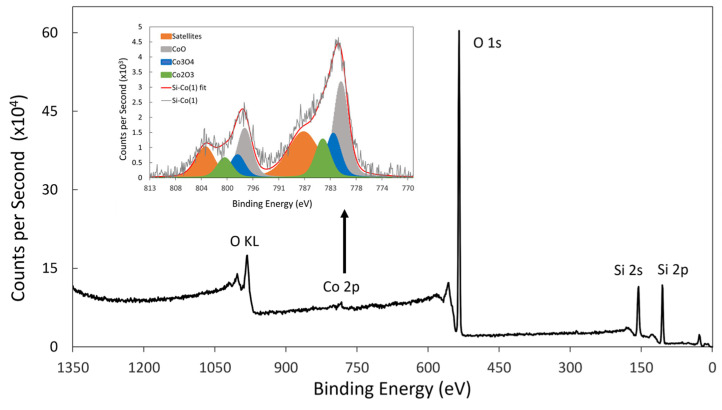
XPS spectra of the Si-Co(1) composite: survey scan and Co *2p* spectrum (inset).

The XPS analysis (Figure 2) provided further insights into the identity of the cobalt species present on the silica surface. Thus, the Co *2p* XPS spectrum for Si-Co(1) showed characteristic peaks for oxidic Co [49,50,51,52], with binding energies of 781.2 eV and 797.0 eV for Co *2p*_3/2_ and Co *2p*_1/2_ electrons, respectively (Figure 2). Satellite peaks for oxidic Co were also observed at 787.1 eV and 803.5 eV. The deconvolution of the Co *2p* XPS spectrum of Si-Co(1) suggested the presence of a mixture of oxidic Co^2+^ and Co^3+^ species on the surface (61.4% and 38.6%, respectively, based on peak areas for CoO (780.9 eV and 796.9 eV), Co_2_O_3_ (783.9 eV and 800.3 eV), and Co_3_O_4_ (782.2 eV and 798.1 eV) from Co *2p* XPS spectrum deconvolution [53]). In contrast, for Si-Co(2) and Si-Co(3) composites, unresolved XPS spectra were observed, presumably due to the comparatively low cobalt surface content of these materials. Therefore, further analysis was performed on the Si-Co(1) composite, which had the highest surface concentration of cobalt oxide species among the three prepared composites.

**Figure 3:**
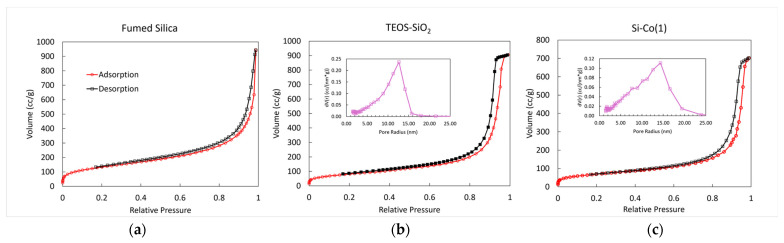
Nitrogen adsorption/desorption isotherms for fumed SiO_2_ (**a**), TES–SiO_2_ (**b**), and Si-Co(1) (**c**), and the pore size distribution (inset) for TES–SiO_2_ and Si-Co(1).

Nitrogen adsorption/desorption isotherms of the starting fumed silica, TES–SiO_2_, and Si-Co(1) composite were recorded at 77 K (Figure 3). Consistent with the nature of unmodified fumed silica, its adsorption/desorption isotherms belong to Type III, characteristic of non-porous materials (Figure 3a). In contrast, nitrogen adsorption/desorption isotherms for TES–SiO_2_ and Si-Co(1) composites indicated mesoporous structures (Type IV isotherms; Figure 3b and Figure 3c, respectively). Thus, the pore radius of the TES–SiO_2_ material was found to be 12.7 nm, with a pore volume of 1.39 cm^3^·g^−1^. Compared to fumed SiO_2_ (395 m^2^·g^−1^) and consistent with the increased pore size, the TES–SiO_2_ sample showed a significantly decreased specific surface area of 285 m^2^·g^−1^, determined by DFT (density functional theory) method [54,55] (the BET (Brunauer–Emmett–Teller) method [56] showed an analogous trend with specific surface areas for TES–SiO_2_ and fumed SiO_2_ of 299 m^2^·g^−1^ and 486 m^2^·g^−1^, respectively). For Si-Co(1), the specific surface area (228 m^2^·g^−1^ and 249 m^2^·g^−1^ by DFT and BET methods, respectively) was found to be smaller compared to that of TES–SiO_2_ and almost half that of the initial fumed SiO_2_ sample (vide supra), suggesting the fusion of silica primary particles upon the synthesis of the composite. This is also reflected in the decreased pore volume of the Si-Co(1) composite measured at saturation (1.07 cm^3^·g^−1^ vs. 1.39 cm^3^·g^−1^ for TES–SiO_2_), whereas the pore radius for Si-Co(1) somewhat increased to 14.4 nm (vs. 12.7 nm for TES–SiO_2_).

The thermogravimetric analysis (TGA) of freshly prepared TES–SiO_2_ and the Si-Co(1) composite (temperature range 30–1000 °C; heating rate 10 °C/min; argon atmosphere) revealed only small amounts of moisture in both samples. The observed mass loss for both samples below 200 °C was approximately 1 wt%, attributed to the loss of physisorbed water (Figures S21 and S22). Overall, both TES-SiO_2_ and Si-Co(1) exhibited high thermal stability, with low weight loss up to 1000 °C, and final residue weights of 98.2% and 96.7%, respectively. To ensure no changes in the moisture content in TES–SiO_2_ and CoO_x_@SiO_2_ composites, all prepared materials were stored under an argon atmosphere.

### 2.2. Catalytic Reduction of 2-Nitrophenol

**Figure 4:**
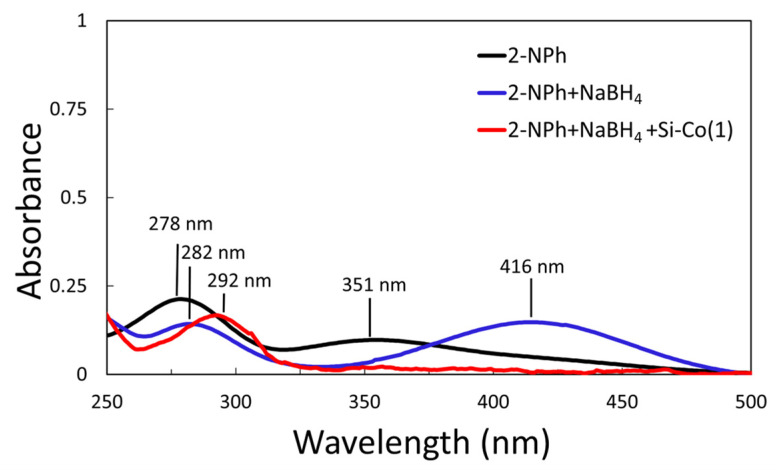
UV-Vis spectra taken from an aqueous solution of 2-NPh before NaBH_4_ addition (black), after NaBH_4_ addition (blue), and 3 min after addition of Si-Co(1) and NaBH_4_ (red).

**Figure 5:**
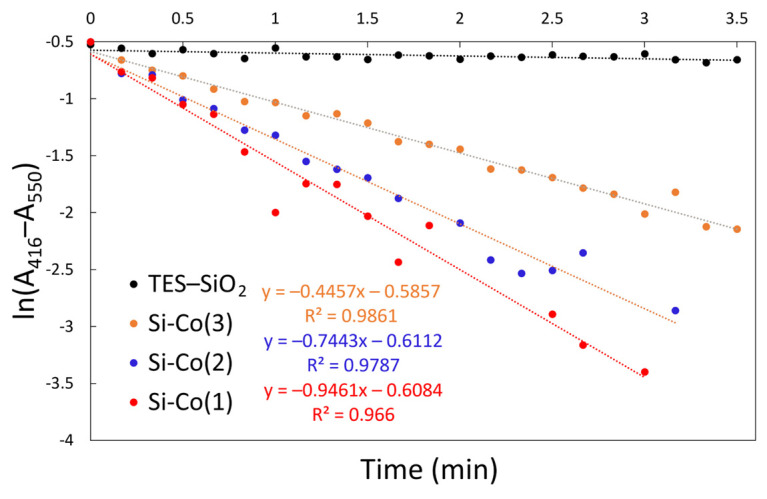
Reaction kinetics analysis for the CoO_x_@SiO_2_-catalyzed reduction of 2-NPh (0.15 mmol·L^−1^) with NaBH_4_ (0.15 mol·L^−1^).

The reduction of 2-NPh with NaBH_4_ was carried out in water using all three prepared composites, Si-Co(1), Si-Co(2), and Si-Co(3). TES–SiO_2_ was also subjected to 2-NPh reduction and showed no catalytic activity, indicating the necessity of the cobalt species in the catalyst material. In contrast, rapid reduction reactions were observed in the presence of CoO_x_@SiO_2_ composites, accompanied by discoloration of the yellow aqueous 2-NPh solution (Figure S24). The ^1^H-NMR analysis of the solid residue after evaporation of water from the reaction mixtures revealed the complete conversion of 2-NPh and the formation of 2-APh as the sole reaction product (Figure S25). The efficiency of the prepared composite pre-catalysts in reducing 2-NPh with NaBH_4_ was assessed using UV-Vis spectroscopy. The reactions were performed in a 10 mm path-length quartz UV-Vis cuvette at a 0.75 μmol 2-NPh scale (C = 0.15 mmol·L^−1^), using 0.75 mmol of NaBH_4_ and 7.7 mg of CoO_x_@SiO_2_ composites, and the progress of the reaction was monitored by UV-Vis spectroscopy. The UV-Vis spectrum of an aqueous solution of 2-NPh showed two distinct absorbance bands at 278 nm and 351 nm [11]. The addition of NaBH_4_ to the aqueous solution of 2-NPh led to the formation of 2-nitrophenolate, resulting in shifts in the absorbance bands to 282 nm and 416 nm, respectively (Figure 4) [57]. In the absence of CoO_x_@SiO_2_ composites or in the presence of TES–SiO_2,_ these absorbance bands remained unaffected. In contrast, the addition of composites Si-Co(1), Si-Co(2), and/or Si-Co(3) facilitated the disappearance of the absorbance band at 416 nm and the shift of the absorbance peak at 282 nm to 291 nm, indicating the formation of 2-APh (Figure 4) [11,58]. Consistent with the highest cobalt loading (vide supra), Si-Co(1) proved to be the most efficient pre-catalyst, resulting in the disappearance of the absorbance band at 416 nm and discoloration of the reaction mixture in 3 min at room temperature (vs. 4.5 min and 7.5 min for composites Si-Co(2) and Si-Co(3), respectively). Continuous monitoring of the absorbance at 416 nm during the CoO_x_@SiO_2_-catalyzed reduction of 2-NPh and plotting the absorbance changes expressed as ln(A_416_–A_550_) (where A_416_ and A_550_ are absorbances at 416 nm and 550 nm, respectively; the absorbance at 550 nm was chosen as a baseline reference point [11], as no features were observed at this wavelength) vs. the reaction time revealed the pseudo-first-order kinetics for the reactions with the rate constants (*k*) for Si-Co(1), Si-Co(2), and Si-Co(3) of 9.5·10^−1^ min^−1^, 7.4·10^−1^ min^−1^, and 4.6·10^−1^ min^−1^, respectively (Figure 5; Table 2, entries 1–3). Notably, these values of the rate constants are comparable to those previously reported for analogous noble metal NPs/SiO_2_-catalized (metal = Pt, Ru, and Au) transformations (found within the range of 1.3·10^−1^ min^−1^–8.6·10^−1^ min^−1^) [11]. At 1.5 μmol loading of 2-NPh (C = 0.5 mmol·L^−1^), keeping the same amounts of all other components (0.75 mmol of NaBH_4_ and 7.7 mg of Si-Co(1)), the disappearance of the 2-NPh absorbance at 416 nm in the Si-Co(1)-catalyzed reaction was observed within 5.5 min at room temperature (*k* = 5.3·10^−1^ min^−1^; Table 2, entry 4; Figures S5 and S6), whereas the same reactions with 0.15 mmol and 15 μmol of NaBH_4_ took 18.5 min (*k* = 1.6·10^−1^ min^−1^; Table 2, entry 5; Figures S3 and S4) and 1.5 h (*k* = 0.4·10^−1^ min^−1^; Table 2, entry 6; Figure S2), respectively. Importantly, no catalytic activity was observed for TES–SiO_2_ (Figure 5, black data points), the bare TES-modified silica without cobalt, under the same experimental conditions as described above. Moreover, under identical reaction conditions (Table 2, entry 1), the reduction of 2-NPh with NaBH_4_ under argon atmosphere using a composite prepared similarly to Si-Co(1) but under anaerobic conditions was found to be faster (*k* = (13.3 ± 1.03)·10^−1^ min^−1^; Figure S27) than the analogous “in air” transformation catalyzed by Si-Co(1) (*k* = (9.5 ± 0.49)·10^−1^ min^−1^). Assuming a lower content of oxidized cobalt vs. low-oxidation-state cobalt compounds in the composite prepared under inert atmosphere, the observed lower reaction rate of Si-Co(1)-catalyzed reduction of 2-NPh under aerobic conditions is likely associated with the activation of the oxidic cobalt species with NaBH_4_ to form catalytically active Co(0) [59,60,61,62] or/and cobalt borides, Co_x_B [63,64] (vide infra).

**Figure 6:**
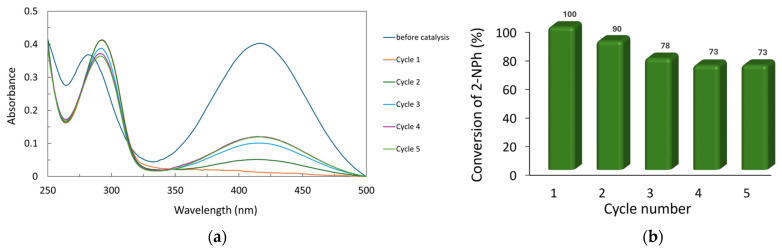
The reusability and recyclability of Si-Co(1) in the reduction of 2-NPh (conditions: 7.7 mg of Si-Co(1), 3 mL of H_2_O, C(2-NPh) = 0.5 mmol·L^−1^, C(NaBH_4_) = 0.25 mol·L^−1^_,_ 5 cycles, each 10 min at room temperature): (**a**) UV-Vis spectra for reduction cycles 1–5, and (**b**) conversion of 2-NPh for reduction cycles 1–5.

The reusability and recyclability of Si-Co(1) in the reduction of 2-NPh were also tested (Figure 6). The reaction was carried out for five consecutive cycles, with the catalyst separated by centrifugation of the reaction mixture after each cycle. Each catalytic cycle was carried out at room temperature for 10 min using the conditions listed in Table 2, entry 4 (7.7 mg of Si-Co(1), 3 mL of H_2_O, 1.5 μmol of 2-NPh, 0.75 mmol of NaBH_4_). The conversion of 2-NPh to 2-APh was calculated based on the UV-Vis spectra recorded after each cycle (Figure 6a). Overall, the catalyst maintained its activity throughout the five 2-NPh reduction cycles, although a decrease in the conversion of 2-NPh to 73–78% was observed after the second cycle (Figure 6b). Analogous conversions of 2-NPh were perceived when the catalyst reusability/recyclability test was repeated on a larger scale using 4.5 μmol of 2-NPh, 2.25 mmol of NaBH_4_, and 23.1 mg of Si-Co(1) in 9 mL of H_2_O (see Figure S28). However, the ICP-OES analysis of the catalyst material recovered after the fifth cycle of the latter experiment showed about 30% of the cobalt loss compared to the starting Si-Co(1).

A possible scenario for triggering the CoO_x_@SiO_2_-catalyzed conversion of 2-NPh to 2-APh is the reduction of the surface Co^2+^/Co^3+^ with NaBH_4_ to Co(0) [59,60,61,62] and/or Co_x_B boride species [63,64,65]. Indeed, vigorous hydrogen evolution was observed during the CoO_x_@SiO_2_-catalyzed reduction of 2-NPh with NaBH_4_, whereas in the absence of 2-NPh, the Si-Co(1) composite was found to effectively mediate the release of H_2_ gas from an aqueous solution of NaBH_4_ [60,61], possibly through intermediate formation of a cobalt boride species as a hydrogen mediator [63,64]. The reduction of Co^2+^/Co^3+^ by NaBH_4_ is likely followed by cobalt-mediated H transfer to the nitro group of 2-NPh to yield 2-APh [66,67]. Indeed, the catalytic activity of supported Co(0) NPs in the reduction of the related 4-NPh by NaBH_4_ has been documented in the literature [27,68]. Moreover, free Co(0) NPs, generated in situ from an aqueous Co(OAc)_2_ solution (30 mol% Co), also proved active in the reduction of 2-NPh with NaBH_4_, showing complete reduction of 2-NPh within 8 min at room temperature (*k* = (7.9 ± 0.21)·10^−1^ min^−1^; Figures S10 and S23). On the other hand, cobalt boride intermediates, Co_x_B, formed by the reaction of surface Co^2+^/Co^3+^ with NaBH_4_ can also mediate the reduction of the nitro group of 2-NPh by NaBH_4_, and such transformations have been previously described in the literature [67]. In addition, for comparison with Si-Co(1), Si-Co(2), and Si-Co(3), a cobalt oxide–silica composite, Si-Co(4) derived from unmodified commercial fumed silica, was prepared, following a slightly modified protocol reported in the literature [69,70]. For this, fumed SiO_2_ (395 m^2^/g) was impregnated with Co(OAc)_2_, and the resulting material was dried at 100 °C for 18 h and calcined at 500 °C for 2 h under aerobic conditions. However, in contrast to Si-Co(1), Si-Co(2), and Si-Co(3), the Si-Co(4) composite showed no catalytic activity in the reduction of 2-NPh with NaBH_4_ (Figure S26), suggesting an important role of the surface silicon hydride groups in the surface stabilization and/or dispersion of cobalt species in TES-SiO_2_-derived composites.

## 3. Materials and Methods

### 3.1. Materials

Commercially available fumed Silica (Sigma-Aldrich, Saint Louis, MO, USA, 395 m^2^/g), Co(OAc)_2_·4H_2_O (Sigma-Aldrich), CoF_2_ (Sigma, Saint Louis, MO, USA), CoCl_2_ (Acros Organics, Geel, Belgium), NaOH (Sigma-Aldrich, >98%), triethoxysilane (Sigma-Aldrich, 95%), glacial acetic acid (Sigma-Aldrich), methanol (Sigma-Aldrich, 99.9% (GC)), nitric acid (Sigma-Aldrich, ACS reagent, 70%), hydrofluoric acid (Sigma-Aldrich; 48 wt.%), 2-nitrophenol (Acros Organics, 99%), NaBH_4_ (Sigma-Aldrich, >98%), and D_2_O (Aldrich; 99.9 atom% D) were used as received without further purification. Deionized (DI) water was obtained using the Thermo Barnstead 7155 reverse osmosis system (Thermo Fisher, Waltham, MA, USA).

### 3.2. Characterization Techniques

FTIR (Fourier transform infrared spectroscopy) was performed using a Nicolet iS10 FT-IR spectrometer (Thermo Fisher, Waltham, MA, USA). SEM (scanning electron microscopy) analysis was carried out using Crossbeam 540 (Carl Zeiss, Jena, Germany). TEM (transmission electron microscopy) was performed using a JEM-1400 Plus (JEOL, Tokyo, Japan). ICP-MS (inductively coupled plasma mass spectrometry) was performed using iCAP RQ ICP-MS (Thermo Scientific, Waltham, MA, USA); conc. HNO_3_ and conc. HF were used for sample digestion. ICP-OES (inductively coupled plasma optical emission spectroscopy) was carried out using Icap 6300 (Thermo Scientific); conc. HNO_3_ and conc. HF were used for sample digestion. Powder XRD (X-ray diffraction) was carried out using SmartLab XRD (Rigaku, Tokyo, Japan). XPS (X-ray photoelectron spectroscopy) was carried out using the Thermo Scientific Nexsa XPS System. The XPS spectra were collected using an AlK*α* source, 400 µm spot size, 0.10 eV step size, and 50 eV band pass energy for regions, and 1.0 eV step size and 100 eV band pass energy for surveys. Thermo Avantage (version 5.9931 build 06755, Thermo Fisher Scientific, Waltham, MA, USA) was used for peak fitting and quantitative data analysis. Binding energies (BEs) were calibrated against the C *1s* line of 284.8 eV [71]. Symmetric line shapes were fitted using a mixed Gaussian/Lorentzian (GL(30)) model. For each Co electronic state, the Co *2p*_1/2_ BE position was restricted to Co *2p*_3/2_ +14.99 eV, and the Co *2p*_1/2_ peak area was restricted to Co *2p*_3/2_ × 0.514. TGA (thermal gravimetric analysis) curves were recorded under argon using a STA 449 F3 Jupiter TGA (Netzsch, Selb, Germany) analyzer at a heating rate of 10 °C/min from 30 to 1000 °C. Nitrogen adsorption–desorption isotherms were recorded using the Autosorb iQ Nitrogen porosimeter (Anton Paar, Graz, Austria). Absorbance was monitored to track the progress of the catalytic reactions using an Evolution 300 UV-Vis spectrophotometer with a double-beam optical design and a Xenon flash lamp (Thermo Fisher Scientific, Waltham, MA, USA). ^1^H-NMR (Proton Nuclear Magnetic Resonance) spectra were recorded in D_2_O (Aldrich, Saint Louis, MO, USA, 99.9 atom % D) using JEOL ECA-500 MHz spectrometer (^1^H: 500 MHz) (JEOL, Tokyo, Japan), the spectra were referenced to residual ^1^H resonance of D_2_O (*δ* 4.79 ppm [72]).

### 3.3. Preparation of Cobalt Oxide–Silica Composites

#### 3.3.1. Synthesis of TES-SiO_2_

Silica was prepared following a slightly modified procedure from the literature [11]. For this, 6 g of commercial fumed silica (Sigma-Aldrich, 395 m^2^/g) was dispersed in an aqueous solution of NaOH (1:0.01 *w*/*w* ratio of silica to NaOH) and stirred at 65 °C for 24 h. The mixture was then cooled down to room temperature, and glacial CH_3_COOH was added to neutralize the unreacted NaOH. The solid was separated by vacuum filtration, then washed three times with 10 mL of methanol, and dried at 100 °C for 48 h. A total of 3.0 g of this material was placed in a round-bottom flask equipped with a reflux condenser and immersed in an oil bath maintained at 90 °C. A solution of triethoxysilane (0.65 mL, 3.5 mmol) in 60 mL of glacial acetic acid was added, and the suspension was stirred for 2 h. After cooling to room temperature, the product, TES–SiO_2_ silica, was collected by vacuum filtration and dried at 100 °C for 48 h (2.5 g).

#### 3.3.2. Synthesis of CoO_x_@SiO_2_

TES–SiO_2_ silica (100 mg) was suspended in 10 mL of 10 mmol·L^−1^ aqueous Co(OAc)_2_ solution. The obtained suspension was stirred at room temperature in the dark for 1 h and then centrifuged (4000 rpm, 10 min). The supernatant was decanted, and the solid was washed three times with 10 mL of DI water, centrifuging after each wash. The obtained solid material of Si-Co(1) was dried in an oven at 100 °C for 18 h (50 mg). CoO_x_@SiO_2_ composites Si-Co(2) (derived from CoCl_2_) and Si-Co(3) (derived from CoF_2_) were prepared analogously to Si-Co(1), yielding 47 mg and 51 mg of products, respectively. The prepared composite materials were stored under an argon atmosphere in a single-station LC Technologies Solutions (Salisbury, MA, USA) inert-atmosphere glovebox.

For comparative studies, an oxidic cobalt–silica composite derived from commercial fumed silica was prepared following a slightly modified procedure from the literature [63,64]. Specifically, fumed silica (100 mg; Sigma-Aldrich, 395 m^2^/g) was suspended in 10 mL of 10 mmol·L^−1^ aqueous Co(OAc)_2_ solution, and the resulting suspension was stirred at room temperature in the dark for 1 h. Then, the mixture was centrifuged (4000 rpm for 10 min), the supernatant was decanted, and the remaining solid was washed three times with 10 mL of DI water, centrifuging after each wash. The obtained solid material was dried in an oven at 100 °C for 18 h. Afterward, the dried sample was placed in a muffle furnace at 500 °C for 2 h, and the solid material of Si-Co(4) (42 mg) was obtained.

### 3.4. CoO_x_@SiO_2_-Catalyzed Reduction of 2-Nitrophenol

The catalytic reduction of 2-NPh was performed directly in a 1 cm path-length quartz UV-Vis cuvette, and the reaction was monitored by UV-Vis spectroscopy, tracking the disappearance of the 2-NPh absorbance band at 416 nm. For this, a stock aqueous solution of 2-NPh (0.3 mmol·L^−1^, 100 mL) was prepared. 2.5 mL of this solution, along with 7.7 mg of CoO_x_@SiO_2_ (all four composite materials, Si-Co(1), Si-Co(2), Si-Co(3), and Si-Co(4) were tested), were placed in a UV-Vis cuvette. A total of 2.5 mL of a freshly prepared aqueous NaBH_4_ solution (0.3 mol·L^−1^) was added to the mixture, and the reaction progress was monitored by UV-Vis using LabSolutions UV-Vis software (version 1.11, 2020 Shimadzu Corporation, Kyoto, Japan), with continuous absorbance measurements at 416 and 550 nm to check the conversion of 2-NPh to 2-APh. The absorbance difference at 416 nm and 550 nm was calculated, and this data was used for further kinetic analysis and to calculate the reaction rate constants (see Figure 5). Si-Co(1)-catalyzed reactions at 1.5 μmol loading of 2-NPh (C = 0.5 mmol·L^−1^) and 0.75 mmol, 0.15 mmol and 15 μmol loading of NaBH_4_ (C = 0.25 mol·L^−1^, 50 mmol·L^−1^ and 5.0 mmol·L^−1^, respectively) were performed analogously, monitoring the disappearance of the 2-NPh absorbance band by UV-Vis spectroscopy (see Figures S2–S6). The latter experiments were done to match the conditions and for the more direct comparison of the catalytic activities of this study to the literature results [11]. The obtained reaction rate constants are summarized in Table 2.

### 3.5. Reusability and Recyclability of Si-Co(1) in the Catalytic Reduction of 2-Nitrophenol

The catalytic reduction of 2-NPh was performed in a centrifuge tube, and the reaction was monitored by UV-Vis spectroscopy, tracking the disappearance of the 2-NPh absorbance band at 416 nm. For this, a stock aqueous solution of 2-NPh (C = 1.0 mmol·L^−1^, 100 mL) was prepared. 1.5 mL of this solution and 7.7 mg of the Si-Co(1) composite were placed in a tube. 1.5 mL of a freshly prepared aqueous NaBH_4_ solution (C = 0.5 mol·L^−1^) was added to the mixture. The resulting reaction mixture was left at room temperature for 10 min, then centrifuged for 5 min at 4000 rpm. The solution was decanted, transferred to a 1 cm path-length quartz UV-Vis cuvette, and analyzed using LabSolutions UV-Vis software (version 1.11, 2020 Shimadzu Corporation) over the range of 200 to 500 nm. Then, new portions of 2-NPh solution (1.5 mL, C = 1.0 mmol·L^−1^) and NaBH_4_ solution (1.5 mL, C = 0.5 mol·L^−1^) were added to the catalyst remaining in the centrifuge tube. The procedure was repeated for five consecutive cycles, with the catalyst separated after each cycle. UV-Vis spectra from the reaction mixture were recorded after each cycle to monitor the conversion of 2-NPh to 2-APh (see Figure 6a). The conversion of 2-NPh (see Figure 6b) was calculated based on the difference between the initial absorbance at 416 nm before the catalysis and the measured absorbance at the same wavelength after a given reaction time for each consecutive cycle. Each resulting UV-Vis spectrum was background corrected at 550 nm, as no features were observed at this wavelength [11]. The catalyst reusability and recyclability test (5 cycles) was repeated on larger scale (4.5 μmol of 2-NPh, 2.25 mmol of NaBH_4_, and 23.1 mg of Si-Co(1) in 9 mL of H_2_O) and showed conversions of 2-NPh, analogous to those observed in the small-scale experiment above (see Figure S28). In the latter experiment, the catalyst material recovered after the 5th cycle was subjected to ICP-OES analysis, which showed a 30% decrease in the concentration of cobalt, compared to Si-Co(1).

### 3.6. Reduction of 2-Nitrophenol Using Co(OAc)_2_ as Pre-Catalyst

A stock aqueous solution of 2-NPh (1.0 mM, 50 mL) was prepared. A total of 1.5 mL of this solution was mixed with 45 µL of 10.0 mM aqueous Co(OAc)_2_ solution in a UV-Vis cuvette. In total, 1.5 mL of a freshly prepared aqueous NaBH_4_ solution (50.0 mM) was added to the mixture, and the reaction progress was monitored by UV-Vis using LabSolutions UV-Vis software, with continuous absorbance measurements at 416 and 550 nm to confirm the full conversion of 2-NPh to 2-APh. The reaction was completed within 8 min at room temperature. The absorbance difference at 416 nm and 550 nm was calculated, and this data was used for further kinetic analysis and to calculate the reaction rate constants (see Figure S10).

## 4. Conclusions

To summarize, CoO_x_@SiO_2_ composites were prepared via the sequential in situ reduction of Co(II) salts (Co(OAc)_2_, CoCl_2,_ and CoF_2_) with TES-modified fumed silica having surface silicon hydride groups, followed by the rapid aerobic oxidation of surface Co(0) to Co^2+^/Co^3+^ oxide species. The prepared CoO_x_@SiO_2_ composites were tested as catalysts for the reduction of 2-NPh with NaBH_4_. Despite the low cobalt content of approximately 0.2 wt%, the composite derived from Co(OAc)_2_ exhibited the highest efficiency in reducing 2-NPh to 2-APh, offering a cost-effective, readily available, non-precious-metal-based system for the remediation of nitrophenols from contaminated water. Moreover, given the simplicity of the developed method and widespread applications of the first-row transition metal oxides in heterogeneous catalysis, environmental remediation, and materials science, this methodology can now be applied to the fabrication of silica-supported composites with other 3D metal oxides. Preparation of such materials and their catalytic activities in environmentally and industrially relevant reduction processes are currently under investigation in our laboratories.

## Figures and Tables

**Table 1:** Cobalt content in Si-Co(1), Si-Co(2), and Si-Co(3) determined by ICP-MS/OES ^1^.

Entry	Composite	mmol·g^−1^	wt%
1	Si-Co(1) ^2^	(2.9 ± 0.5)·10^−2^	(1.7 ± 0.3)·10^−1^
2	Si-Co(2)	(2.2 ± 0.2)·10^−2^	(1.3 ± 0.1)·10^−1^
3	Si-Co(3)	(2.0 ± 0.3)·10^−2^	(1.1 ± 0.2)·10^−1^

^1^ If not noted otherwise, ICP-MS analysis was performed (an average Co concentration after three consecutive runs is shown). ^2^ Determined by ICP-MS and ICP-OES (an average value of three ICP-MS and one ICP-OES runs is shown).

**Table 2:** Reaction times (*t*) and rate constants (*k*) of 7.7 mg CoO_x_@SiO_2_-catalyzed reduction of 2-NPh with NaBH_4_.

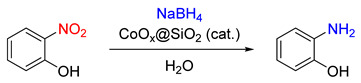							
Entry	Cat.	C(2-NPh)	n(2-NPh)	C(NaBH_4_)	n(NaBH_4_)	*t*, min	*k*, min^−1^
1	Si-Co(1) ^1^	0.15 mmol·L^−1^	0.75 μmol	0.15 mol·L^−1^	0.75 mmol	3	(9.5 ± 0.49)·10^−1^
2	Si-Co(2) ^1^	0.15 mmol·L^−1^	0.75 μmol	0.15 mol·L^−1^	0.75 mmol	4.5	(7.4 ± 0.28)·10^−1^
3	Si-Co(3) ^1^	0.15 mmol·L^−1^	0.75 μmol	0.15 mol·L^−1^	0.75 mmol	7.5	(4.6 ± 0.12)·10^−1^
4	Si-Co(1) ^2^	0.5 mmol·L^−1^	1.5 μmol	0.25 mol·L^−1^	0.75 mmol	5.5	(5.3 ± 0.15)·10^−1^
5	Si-Co(1) ^2^	0.5 mmol·L^−1^	1.5 μmol	50 mmol·L^−1^	0.15 mmol	18.5	(1.6 ± 0.02)·10^−1^
6	Si-Co(1) ^2^	0.5 mmol·L^−1^	1.5 μmol	5 mmol·L^−1^	15 μmol	90	(0.4 ± 0.002)·10^−1^

^1^ 7.7 mg of CoO_x_@SiO_2_ in 5 mL of H_2_O. ^2^ 7.7 mg of CoO_x_@SiO_2_ in 3 mL of H_2_O.

## Data Availability

Data supporting the reported results are available in the Supplementary Materials for this article and from the authors.

## Supplementary Materials

The following supporting information can be downloaded at: https://www.mdpi.com/article/10.3390/molecules31132282/s1, Figure S1. UV-Vis spectra taken from an aqueous solution of 2-NPh before NaBH_4_ addition (black), after NaBH_4_ addition (blue), and 3 min after addition of Si-Co(1) and NaBH_4_ (red). Figure S2. Reaction kinetics analysis for the Si-Co(1)-catalyzed reduction of 2-NPh (mmol·L^−1^) with NaBH_4_ (5.0 mmol·L^−1^). Figure S3. Reaction kinetics analysis for the Si-Co(1)-catalyzed reduction of 2-NPh (0.5 mmol·L^−1^) with NaBH_4_ (50 mmol·L^−1^). Figure S4. Reaction kinetics analysis with linear regression analysis for the Si-Co(1)-catalyzed reduction of 2-NPh (0.5 mmol·L^−1^) with NaBH_4_ (50 mmol·L^−1^). Figure S5. Reaction kinetics analysis for the Si-Co(1)-catalyzed reduction of 2-NPh (0.5 mmol·L^−1^) with NaBH_4_ (0.25 mol·L^−1^). Figure S6. Reaction kinetics analysis with linear regression analysis for the Si-Co(1)-catalyzed reduction of 2-NPh (0.5 mmol·L^−1^) with NaBH_4_ (0.25 mol·L^−1^). Figure S7. Reaction kinetics analysis with linear regression analysis for the Si-Co(1)-catalyzed reduction of 2-NPh (0.15 mmol·L^−1^) with NaBH_4_ (0.15 mol·L^−1^). Figure S8. Reaction kinetics analysis with linear regression analysis for the Si-Co(2)-catalyzed reduction of 2-NPh (0.15 mmol·L^−1^) with NaBH_4_ (0.15 mol·L^−1^). Figure S9. Reaction kinetics analysis with linear regression analysis for the Si-Co(3)-catalyzed reduction of 2-NPh (0.15 mmol·L^−1^) with NaBH_4_ (0.15 mmol·L^−1^). Figure S10. Reaction kinetics analysis with linear regression analysis for the cobalt (II) acetate (30 mol% Co) catalyzed reduction of 2-NPh (0.5 mmol·L^−1^) with NaBH_4_ (50 mmol·L^−1^). Figure S11. Powder XRD spectrum for Si-Co(1). Figure S12. SEM-EDS analysis for TES–SiO_2_. Figure S13. SEM-EDS analysis for Si-Co(1). Figure S14. SEM-EDS analysis for Si-Co(2). Figure S15. SEM-EDS analysis for Si-Co(3). Figure S16. SEM images of TES–SiO_2_. Figure S17. SEM images of Si-Co(1). Figure S18. SEM images of Si-Co(2). Figure S19. SEM images of Si-Co(3). Figure S20. TEM images of fumed SiO_2_ (a), TES–SiO_2_ (b), and Si-Co(1) (c). Figure S21. TGA curve for TES–SiO_2_. Figure S22. TGA curve for Si-Co(1). Figure S23. Reaction mixture of 2-NPh with NaBH_4_ and cobalt (II) acetate as catalysts (30 mol% Co) at the beginning (left) and the end (after 8 min at room temperature) of the reaction (right). Figure S24. Reaction mixture of 2-NPh with NaBH_4_ and Si-Co(1) as catalysts at the beginning (left) and the end (after 3 min at room temperature) of the reaction (right). Figure S25. ^1^H-NMR spectra (in D_2_O) of 2-NPh (top) and the solid residue after Si-Co(1)-catalyzed reduction of 2-NPh with NaBH_4_, showing complete conversion of 2-NPh and formation of 2-APh (bottom). Figure S26. UV-Vis spectra taken from an aqueous solution of 2-NPh after NaBH_4_ addition (black), and 10 min after addition of Si-Co(4) and NaBH_4_ (red). Figure S27. Reaction kinetics analysis with linear regression analysis for the reduction of 2-NPh (0.15 mmol·L^−1^) with NaBH_4_ (0.15 mol·L^−1^) performed under argon atmosphere (in degassed water) using a composite prepared analogously to Si-Co(1) but under anaerobic conditions. Figure S28. The reusability and recyclability of Si-Co(1) in the reduction of 2-NPh (conditions: 23.1 mg of Si-Co(1), 9 mL of H_2_O, C(2-NPh) = 0.5 mmol·L^−1^, C(NaBH_4_) = 0.25 mol·L^−1^, 5 cycles, each cycle is 10 min at room temperature (conversions of 2-NPh: cycle 1—100%, cycle 2—87±5%, cycle 3—78 ± 1%, cycle 4—71 ± 3%, cycle 5—68 ± 7%). Note that concentrations of 2-NPh and NaBH_4_ did not change from the experiment described in Figure 6, the volume of each solution was increased three times along with the loading of catalyst. Additional analytical data supporting the manuscript, including UV-Vis spectra, ^1^H-NMR spectra, and kinetics analysis plots for CoO_x_@SiO_2_-catalyzed reduction of 2-NPh, XRD spectrum of Si-Co(1), SEM-EDS analysis and SEM images of TES-SiO_2_ and CoO_x_@SiO_2_ composites, TGA of TES-SiO_2_ and Si-Co(1), and the photographs of reaction mixtures before and after Si-Co(1)- and Co(OAc)_2_-mediated reduction of 2-NPh, TEM images of fumed SiO_2_, TES-SiO_2_, and Si-Co(1).
