# Supplementary material for: Fumed Silica-Derived CoOx@SiO2 Composites for Catalytic Reduction of 2-Nitrophenol
Source: Molecules. 2026 Jun 30;31(13):2282. doi: 10.3390/molecules31132282 (PMC13363632; doi:10.3390/molecules31132282)
Supplement: Supplementary file 1 [file molecules-31-02282-s001.zip › molecules-4368394-supplementary.pdf]

Supporting Information for

Fumed Silica-Derived CoOx@SiO2 Composites for Catalytic Reduction of 2-Nitrophenol

Amina Zharkenova [1], Aigerim Galyamova [1], Vassilis J. Inglezakis [2,3], Andrey Y. Khalimon [1,*]

[1] Department of Chemistry, School of Sciences and Humanities, Nazarbayev University, 53 Kabanbay Batyr Avenue, Astana 010000, Kazakhstan; amina.zharkenova@nu.edu.kz (A.Z.); aigerim.galyamova@nu.edu.kz (A.G.)

[2] Department of Chemical and Process Engineering, University of Strathclyde, Glasgow G1 1XJ, UK; vasileios.inglezakis@strath.ac.uk

[3] Department of Chemical and Materials Engineering, School of Engineering and Digital Sciences, Nazarbayev University, 53 Kabanbay Batyr Avenue, Astana 010000, Kazakhstan

* Correspondence: andrey.khalimon@nu.edu.kz

Table of Contents

Figure S1. UV-Vis spectra taken from an aqueous solution of 2-NPh before $NaBH_4$ addition (black), after $NaBH_4$ addition (blue), and 3 min after addition of Si-Co(1) and $NaBH_4$ (red)	S3
Figure S2. Reaction kinetics analysis for the Si-Co(1)-catalyzed reduction of 2-NPh (0.5 mM) with $NaBH_4$ (5.0 mM)	S3
Figure S3. Reaction kinetics analysis for the Si-Co(1)-catalyzed reduction of 2-NPh (0.5 mM) with $NaBH_4$ (50 mM).	S4
Figure S4. Reaction kinetics analysis with linear regression analysis for the Si-Co(1)-catalyzed reduction of 2-NPh (0.5 mM) with $NaBH_4$ (50 mM).	S4
Figure S5. Reaction kinetics analysis for the Si-Co(1)-catalyzed reduction of 2-NPh (0.5 mM) with $NaBH_4$ (0.25 M)	S5
Figure S6. Reaction kinetics analysis with linear regression analysis for the Si-Co(1)-catalyzed reduction of 2-NPh (0.5 mM) with $NaBH_4$ (0.25 M)	S5
Figure S7. Reaction kinetics analysis with linear regression analysis for the Si-Co(1)-catalyzed reduction of 2-NPh (0.15 mM) with $NaBH_4$ (0.15 M)	S6
Figure S8. Reaction kinetics analysis with linear regression analysis for the Si-Co(2)-catalyzed reduction of 2-NPh (0.15 mM) with $NaBH_4$ (0.15 M)	S6
Figure S9. Reaction kinetics analysis with linear regression analysis for the Si-Co(3)-catalyzed reduction of 2-NPh (0.15 mM) with $NaBH_4$ (0.15 M)	S7
Figure S10. Reaction kinetics analysis with linear regression analysis for the cobalt (II) acetate (30 mol% Co) catalyzed reduction of 2-NPh (0.5 mM) with $NaBH_4$ (50 mM)	S7
Figure S11. Powder XRD spectrum for Si-Co(1)	S8
Figure S12. SEM-EDS analysis for TES-SiO_2	S9
Figure S13. SEM-EDS analysis for Si-Co(1)	S10
Figure S14. SEM-EDS analysis for Si-Co(2)	S11
Figure S15. SEM-EDS analysis for Si-Co(3)	S12
Figure S16. SEM images of TES-SiO_2	S13
Figure S17. SEM images of Si-Co(1)	S14
Figure S18. SEM images of Si-Co(2)	S15
Figure S19. SEM images of Si-Co(3)	S16
Figure S20. TEM images of fumed SiO_2 (**a**), TES-SiO2 (**b**), and Si-Co(1) (**c**)	S17
Figure S21. TGA curve for TES-SiO_2.	S18
Figure S22. TGA curve for Si-Co(1)	S18
Figure S23. Reaction mixture of 2-NPh with $NaBH_4$ and cobalt (II) acetate as catalysts (30 mol% Co) at the beginning (left) and the end (after 8 min at room temperature) of the reaction (right).	S19
Figure S24. Reaction mixture of 2-NPh with $NaBH_4$ and Si-Co(1) as catalysts at the beginning (left) and the end (after 3 min at room temperature) of the reaction (right)	S19
Figure S25. ^{1}H-NMR spectra (in D_2O) of 2-NPh (top) and the solid residue after Si-Co(1)-catalyzed reduction of 2-NPh with $NaBH_4$, showing complete conversion of 2-NPh and formation of 2-APh (bottom)	S20
Figure S26. UV-Vis spectra taken from an aqueous solution of 2-NPh after $NaBH_4$ addition (black), and 10 min after addition of Si-Co(4) and $NaBH_4$ (red)	S20
Figure S27. Reaction kinetics analysis with linear regression analysis for the reduction of 2-NPh (0.15 mM) with $NaBH_4$ (0.15 M) performed under argon atmosphere (in degassed water) using a composite prepared analogously to Si-Co(1) but under anaerobic conditions.	S21
Figure S28. The reusability and recyclability of Si-Co(1) in the reduction of 2-NPh (conditions: 23.1 mg of Si-Co(1), 9 mL of H_2O, C(2-NPh) = 0.5 mmol·L^{-1}, C($NaBH_4$) = 0.25 mol·L^{-1}, 5 cycles, each cycle is 10 min at room temperature (conversions of 2-NPh: cycle 1 – 100%, cycle 2 – 87±5%, cycle 3 – 78±1%, cycle 4 – 71±3%, cycle 5 – 68±7%). Note that concentrations of 2-NPh and $NaBH_4$ did not change from the experiment described in Figure 6, the volume of each solution was increased three times along with the loading of catalyst.	S22

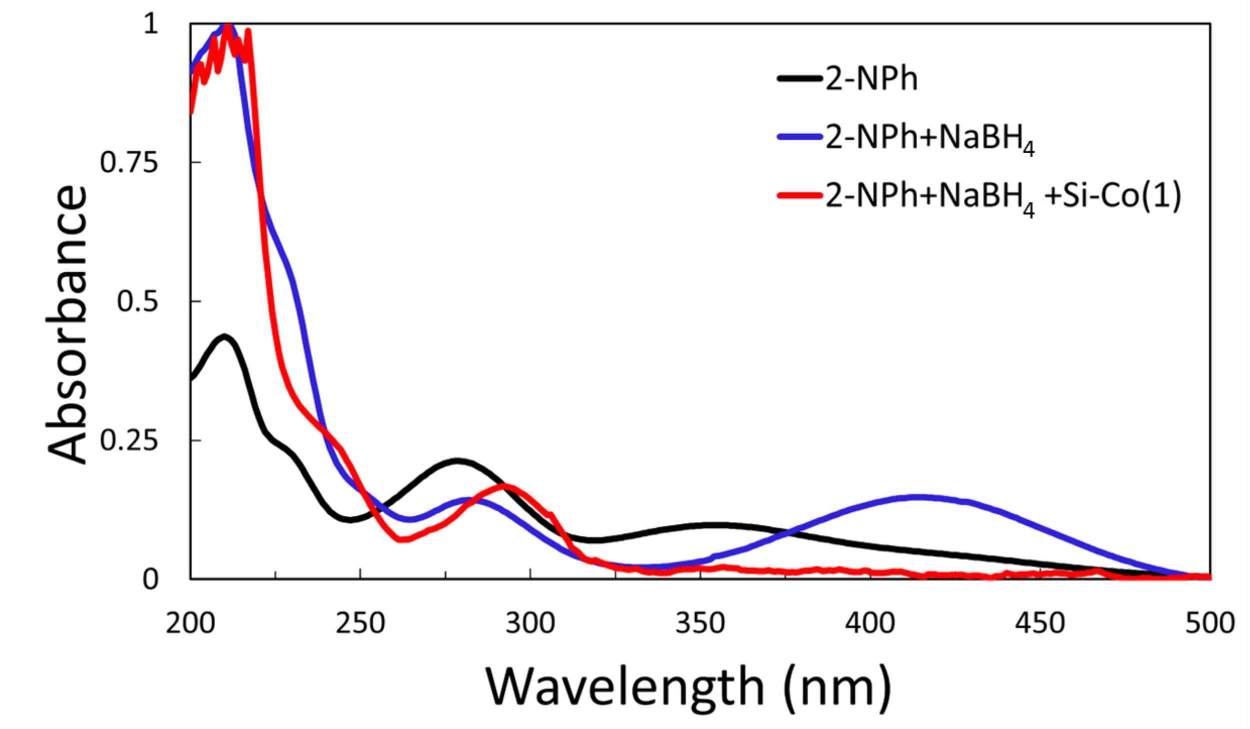

Figure S1. UV-Vis spectra taken from an aqueous solution of 2-NPh before $NaBH_4$ addition (black), after $NaBH_4$ addition (blue), and 3 min after addition of Si-Co(1) and $NaBH_4$ (red).

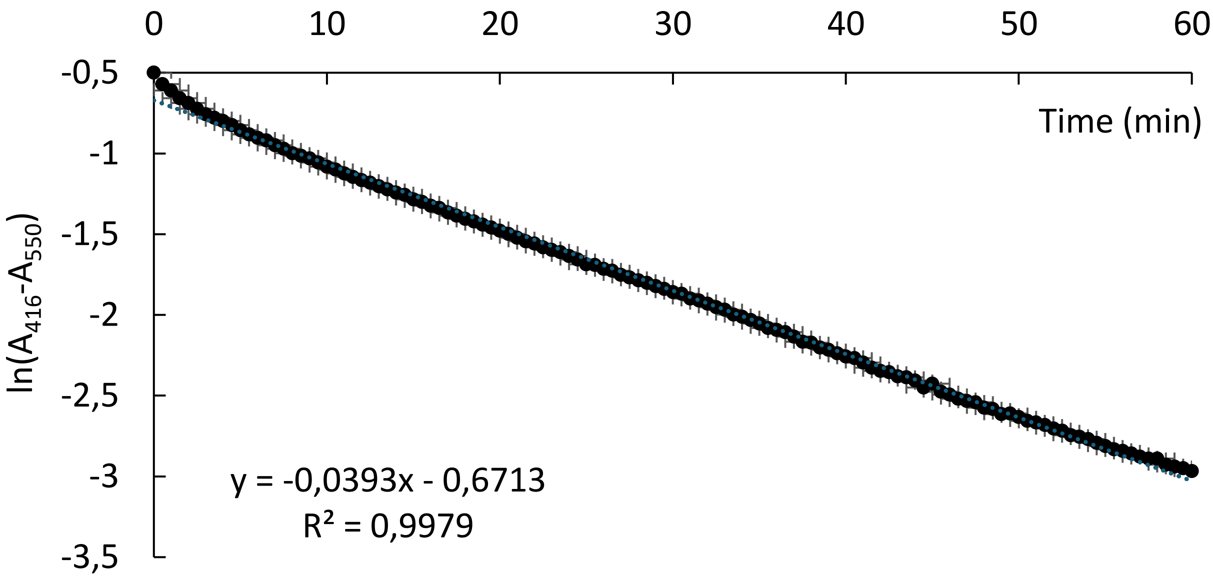

Figure S2. Reaction kinetics analysis for the Si-Co(1)-catalyzed reduction of 2-NPh (0.5 mM) with $NaBH_4$ (5.0 mM).

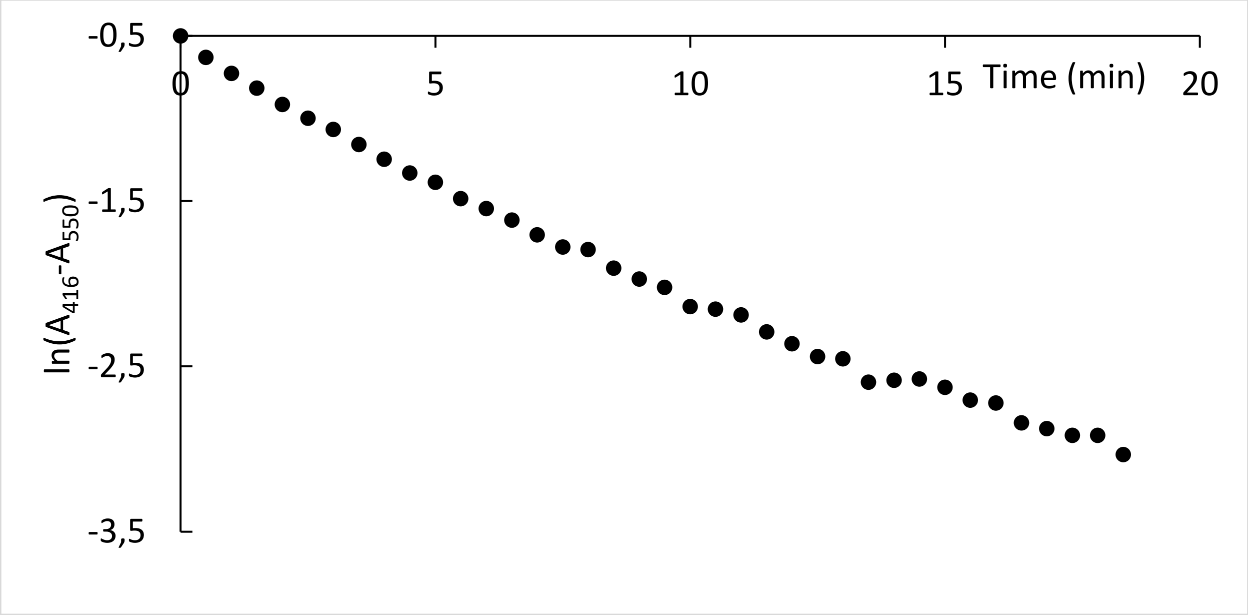

Figure S3. Reaction kinetics analysis for the Si-Co(1)-catalyzed reduction of 2-NPh (0.5 mM) with $NaBH_4$ (50 mM).

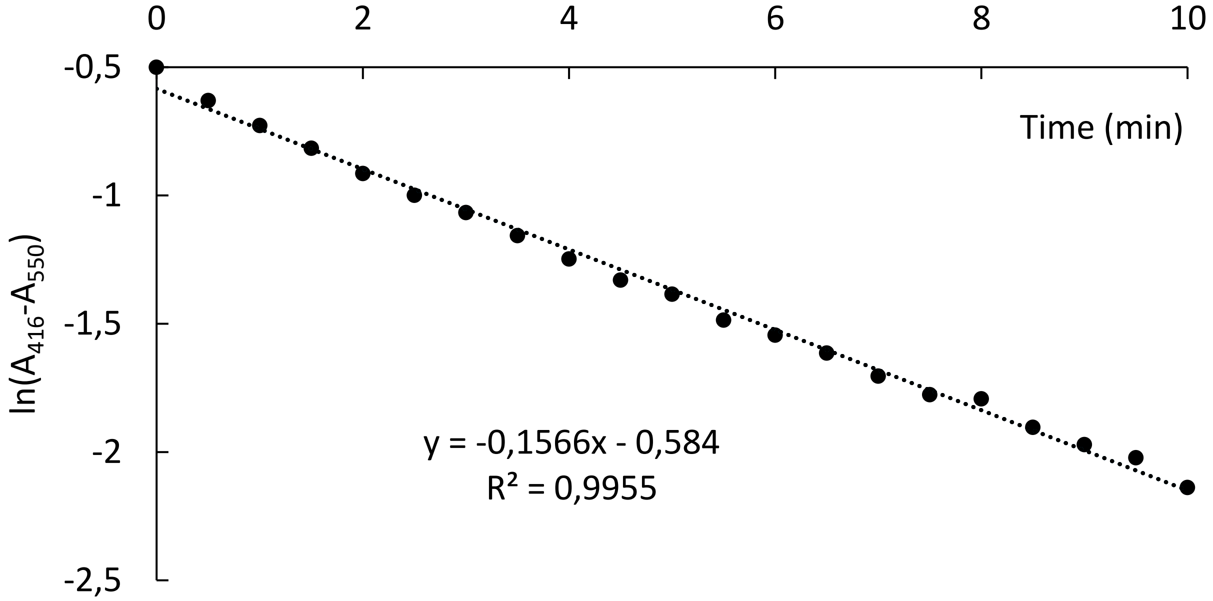

Figure S4. Reaction kinetics analysis with linear regression analysis for the Si-Co(1)-catalyzed reduction of 2-NPh (0.5 mM) with $NaBH_4$ (50 mM).

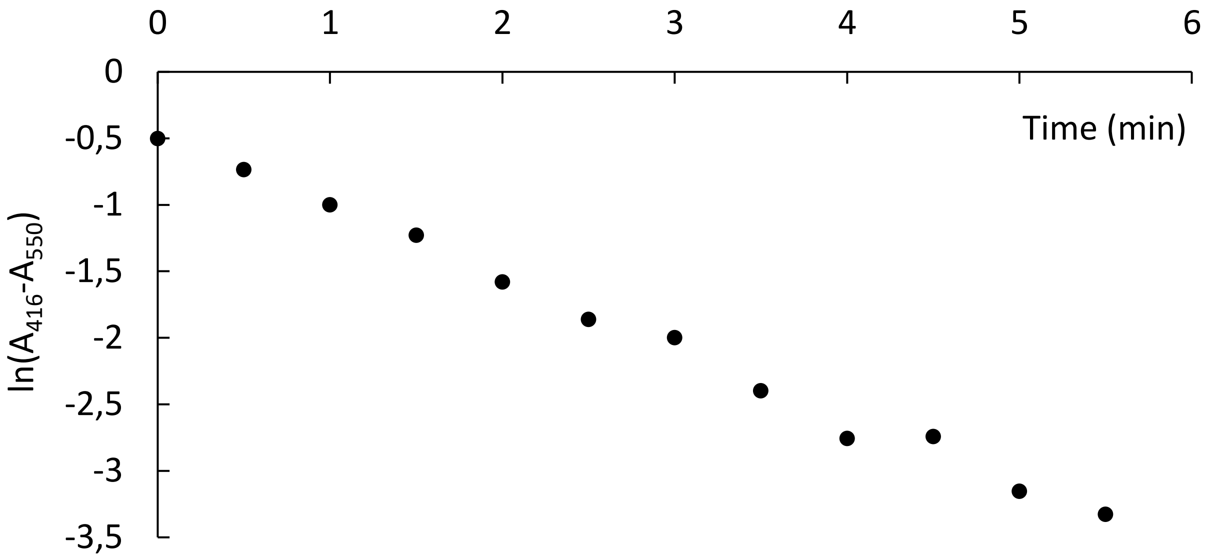

Figure S5. Reaction kinetics analysis for the Si-Co(1)-catalyzed reduction of 2-NPh (0.5 mM) with $NaBH_4$ (0.25 M).

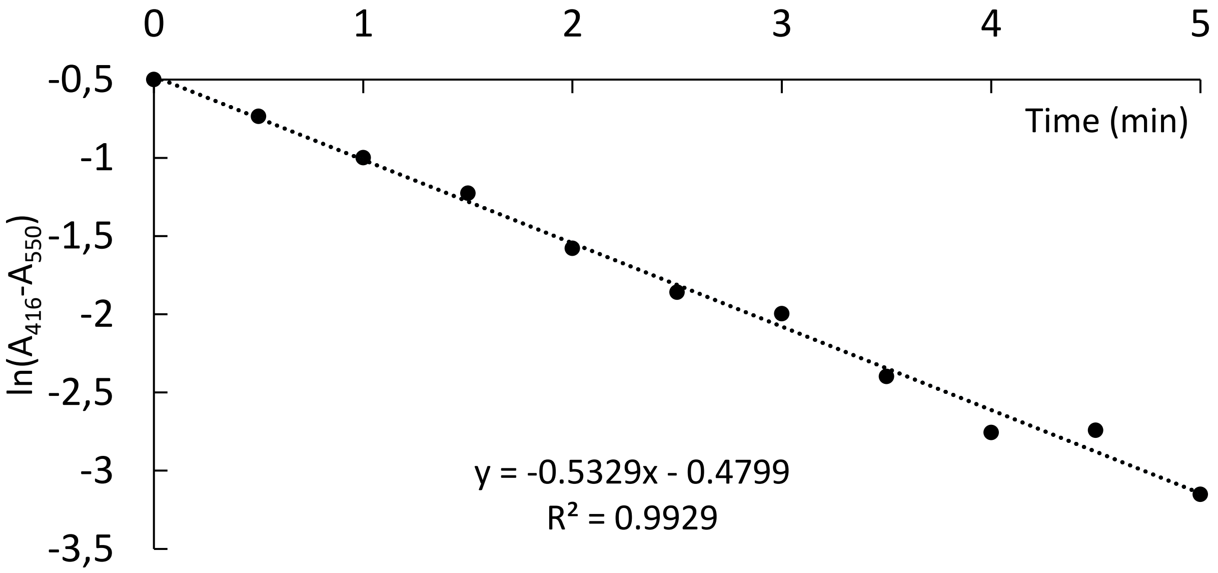

Figure S6. Reaction kinetics analysis with linear regression analysis for the Si-Co(1)-catalyzed reduction of 2-NPh (0.5 mM) with $NaBH_4$ (0.25 M).

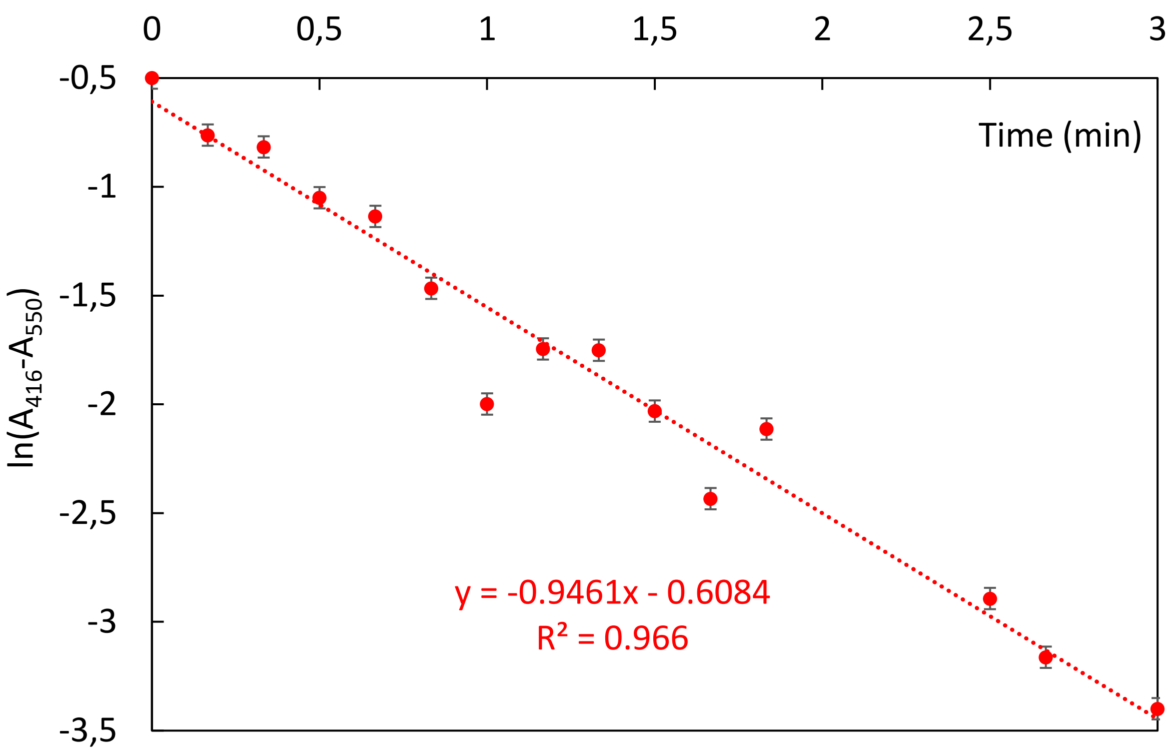

Figure S7. Reaction kinetics analysis with linear regression analysis for the Si-Co(1)-catalyzed reduction of 2-NPh (0.15 mM) with $NaBH_4$ (0.15 M).

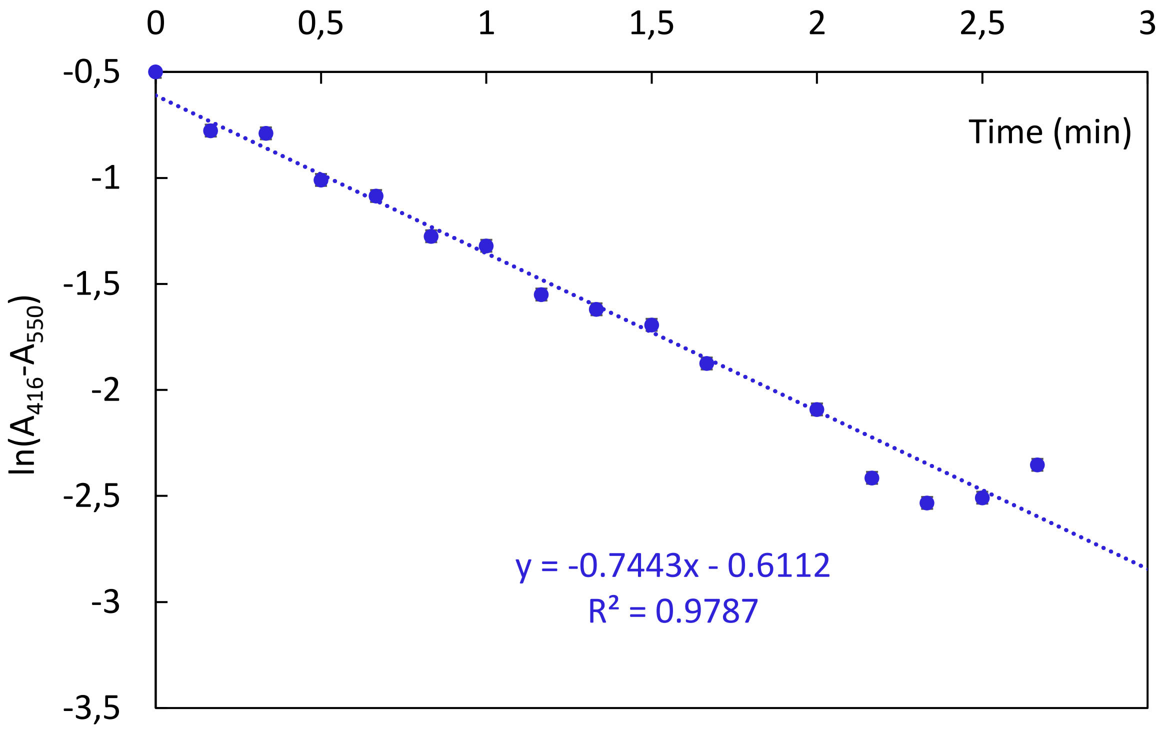

Figure S8. Reaction kinetics analysis with linear regression analysis for the Si-Co(2)-catalyzed reduction of 2-NPh (0.15 mM) with $NaBH_4$ (0.15 M).

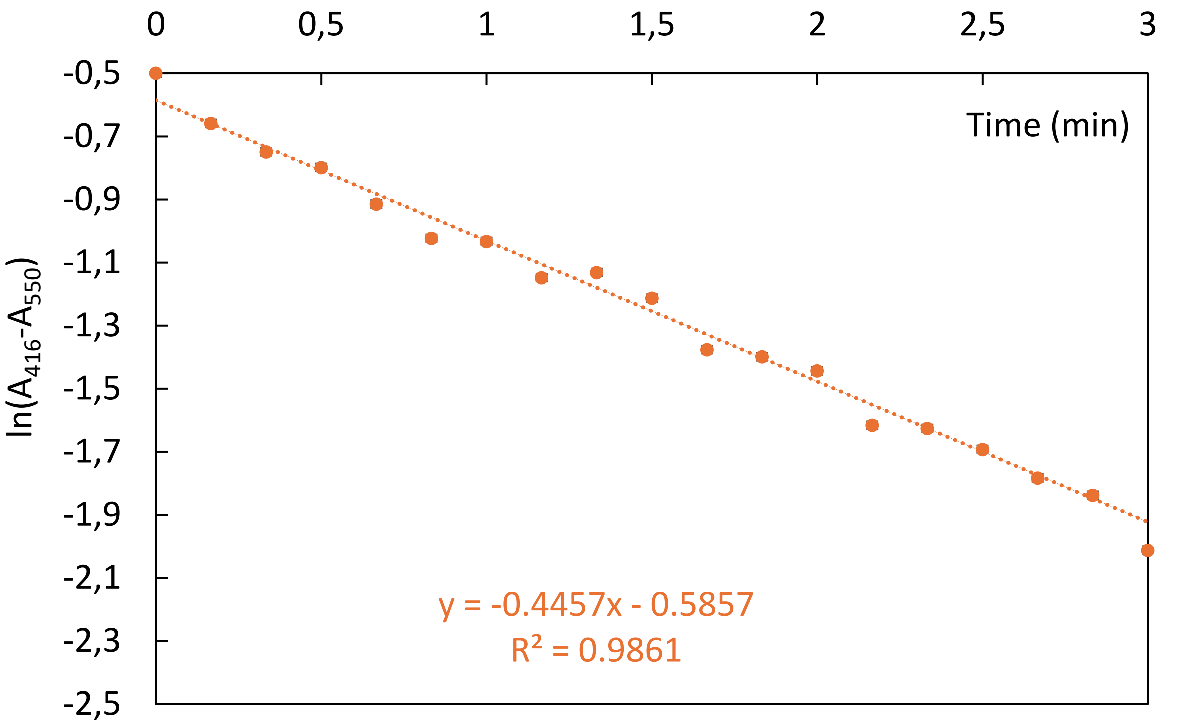

Figure S9. Reaction kinetics analysis with linear regression analysis for the Si-Co(3)-catalyzed reduction of 2-NPh (0.15 mM) with $NaBH_4$ (0.15 M).

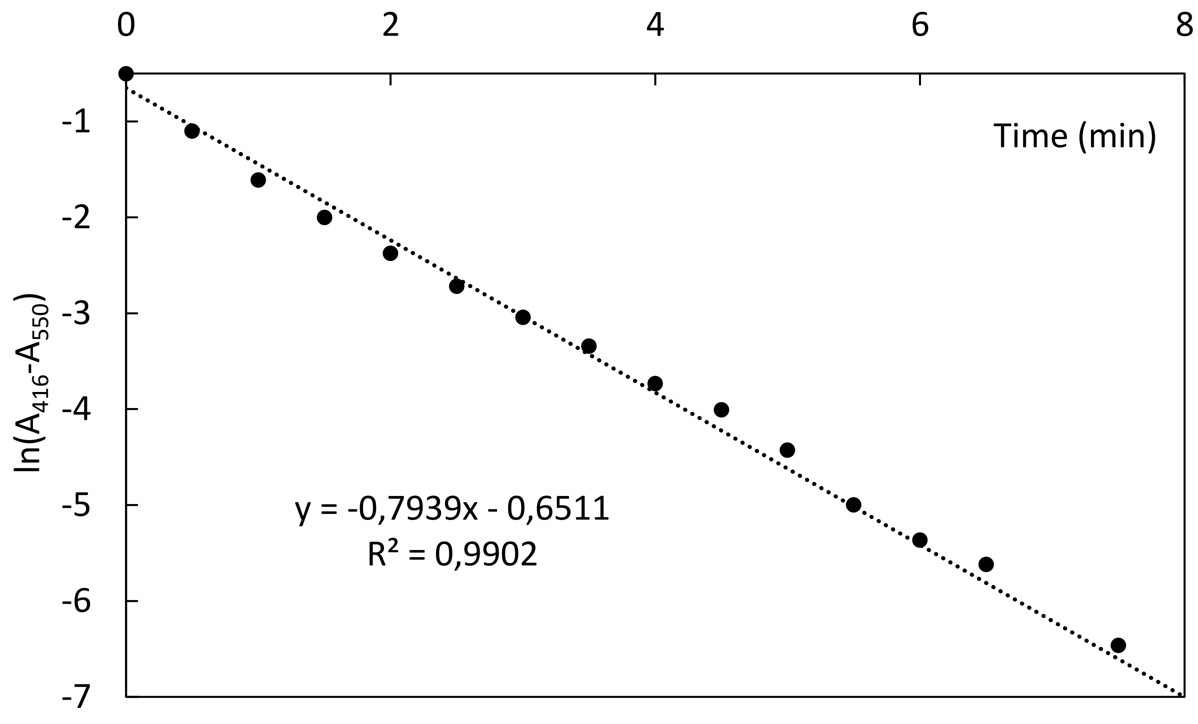

Figure S10. Reaction kinetics analysis with linear regression analysis for the cobalt (II) acetate (30 mol% Co) catalyzed reduction of 2-NPh (0.5 mM) with $NaBH_4$ (50 mM).

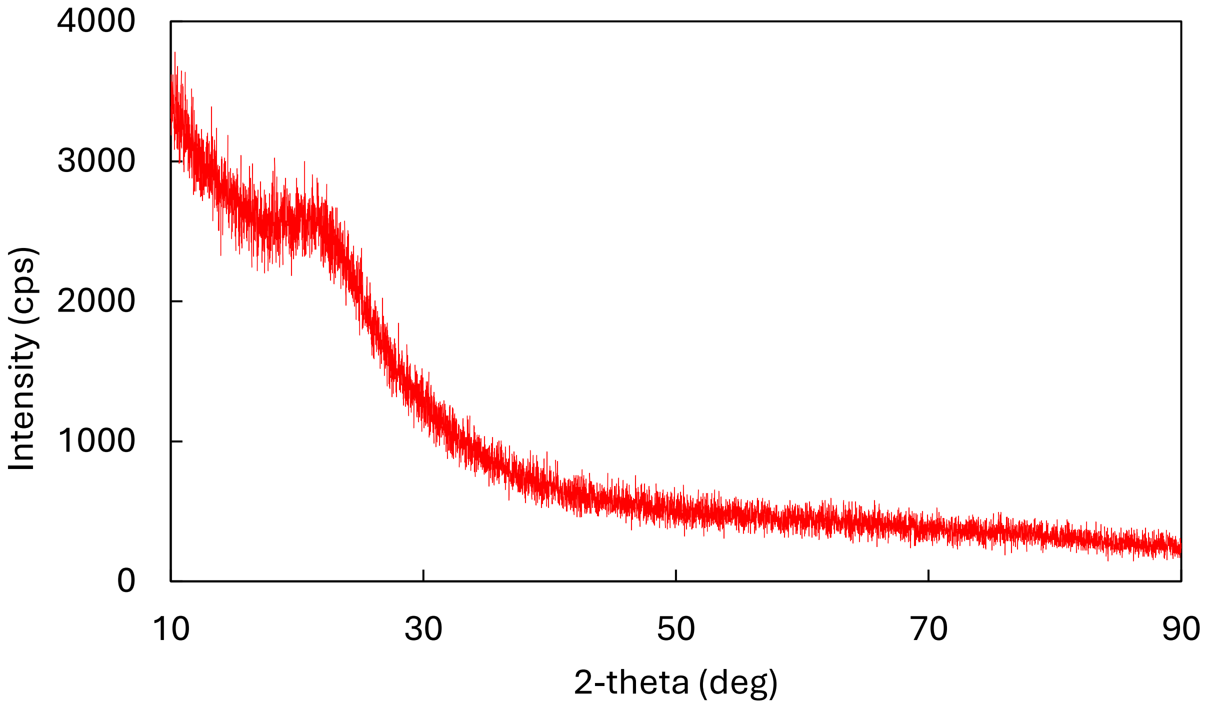

Figure S11. Powder XRD spectrum for Si-Co(1).

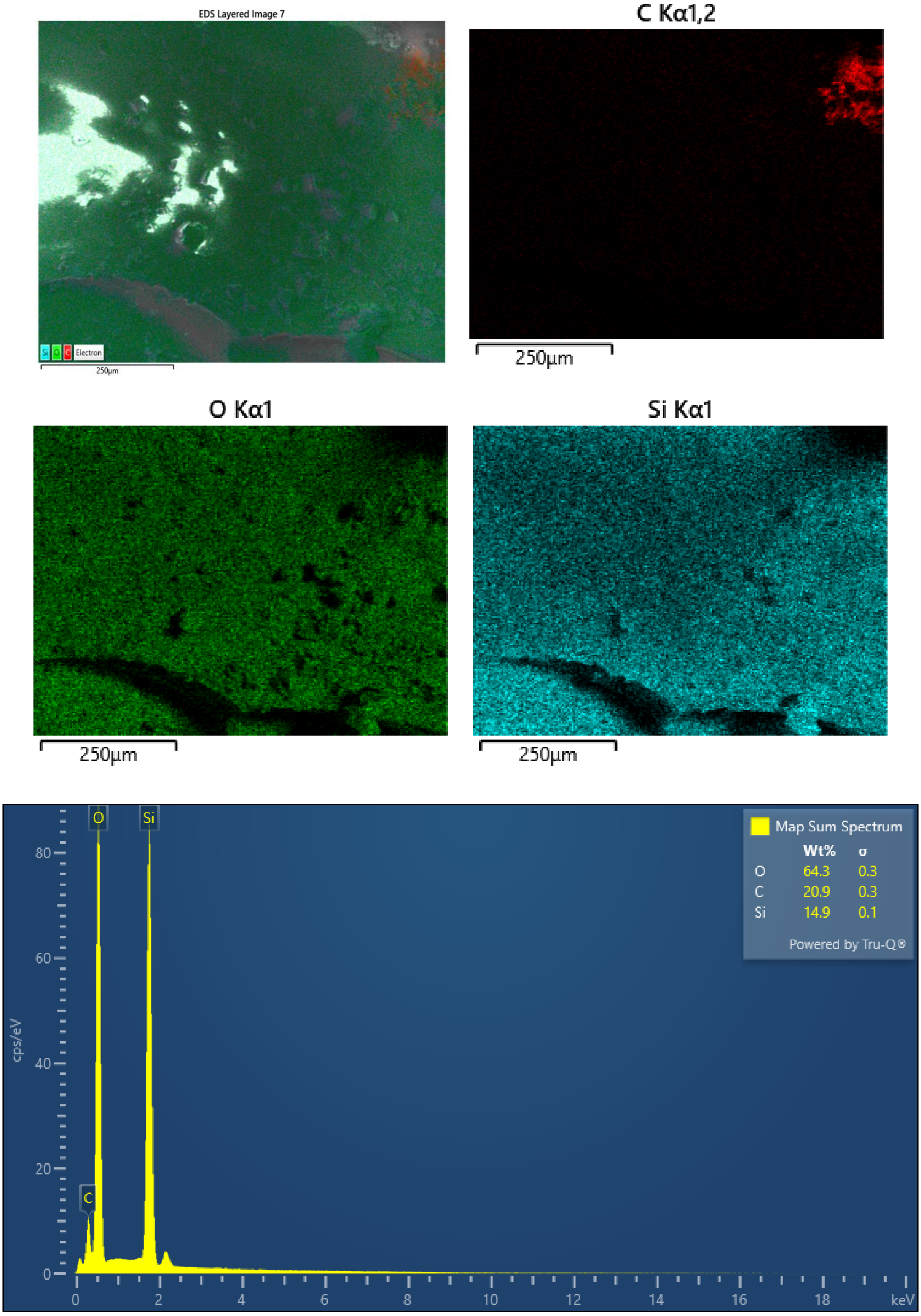

Figure S12. SEM-EDS analysis for TES-SiO_2.

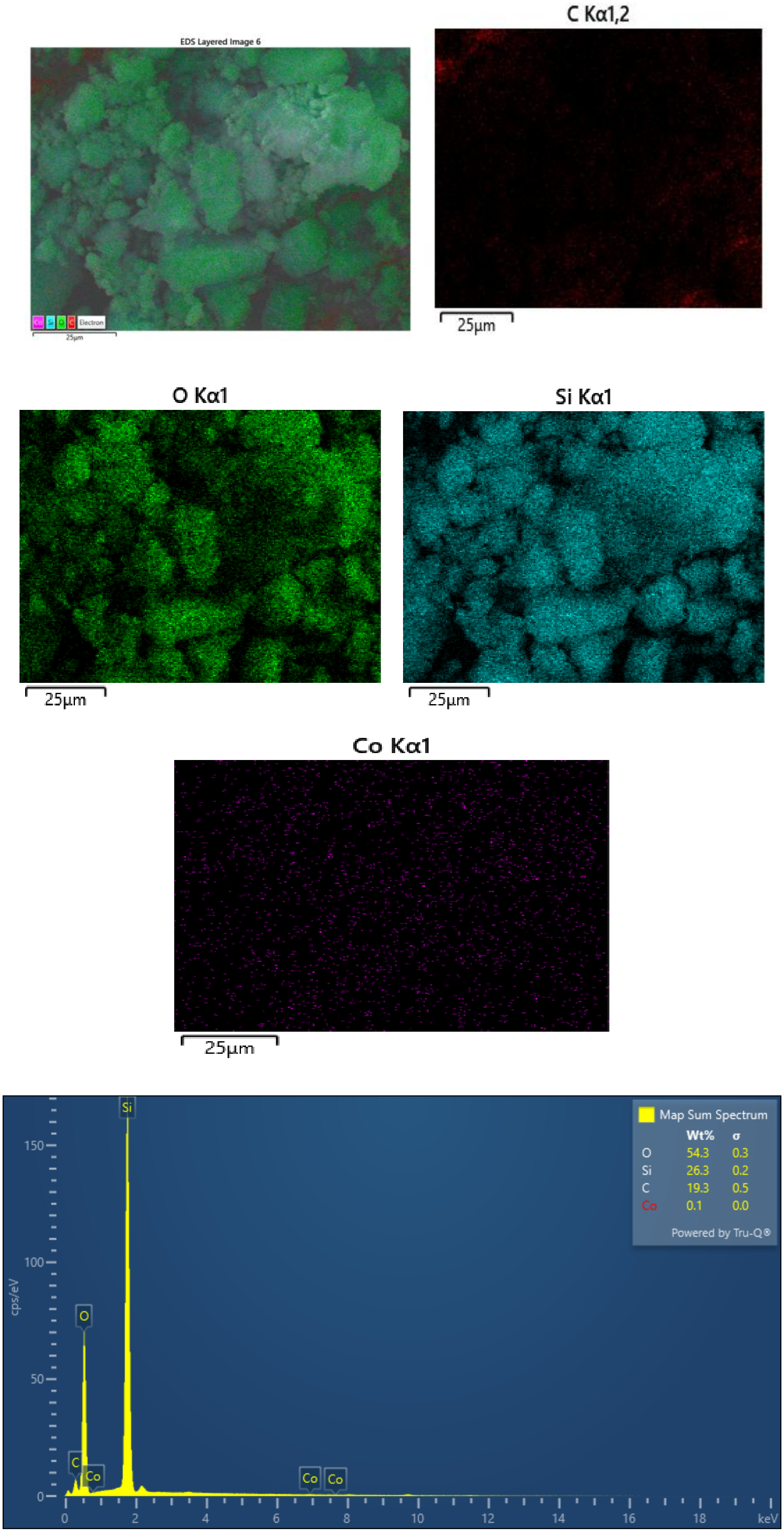

Figure S13. SEM-EDS analysis for Si-Co(1).

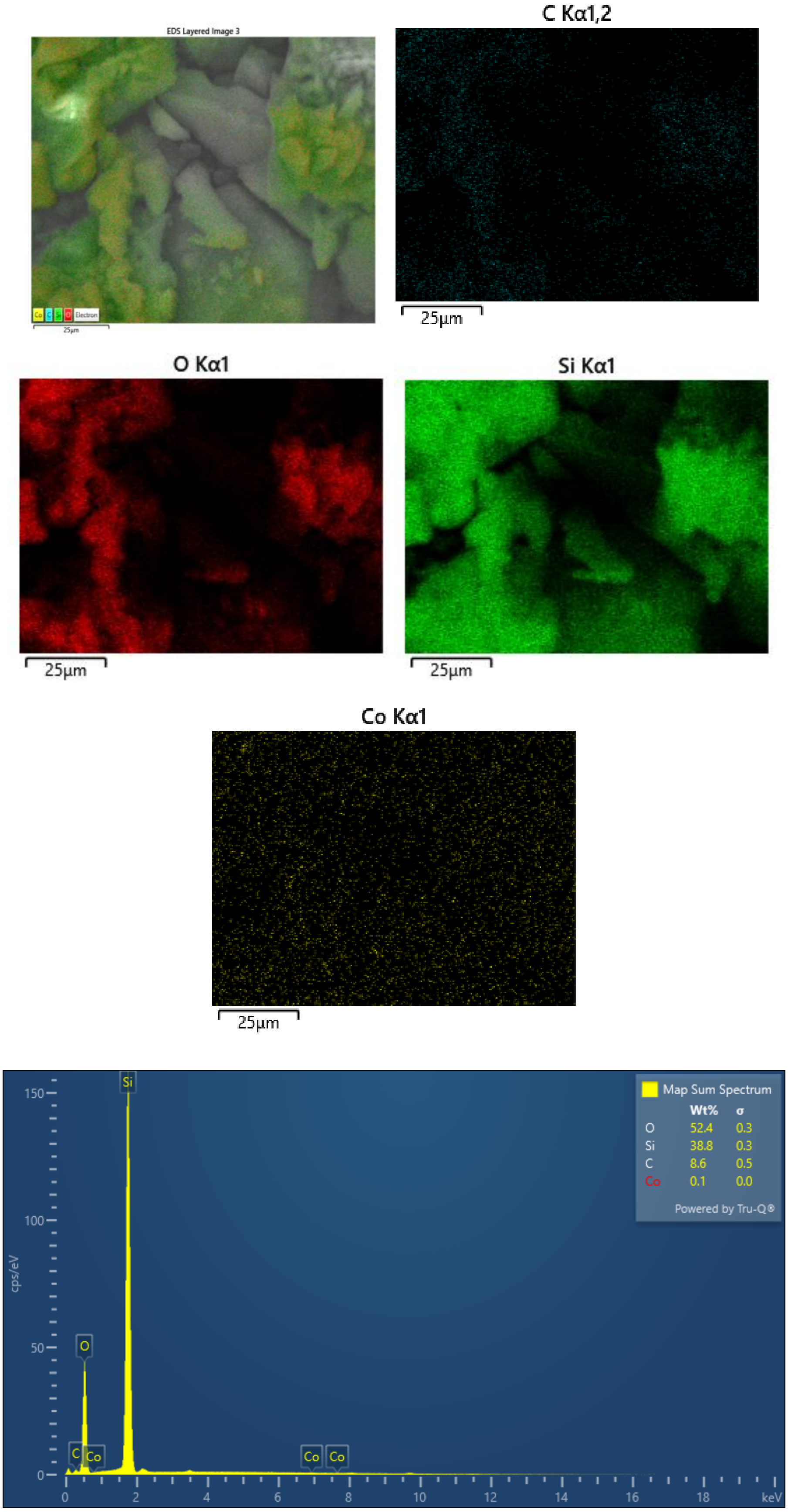

Figure S14. SEM-EDS analysis for Si-Co(2).

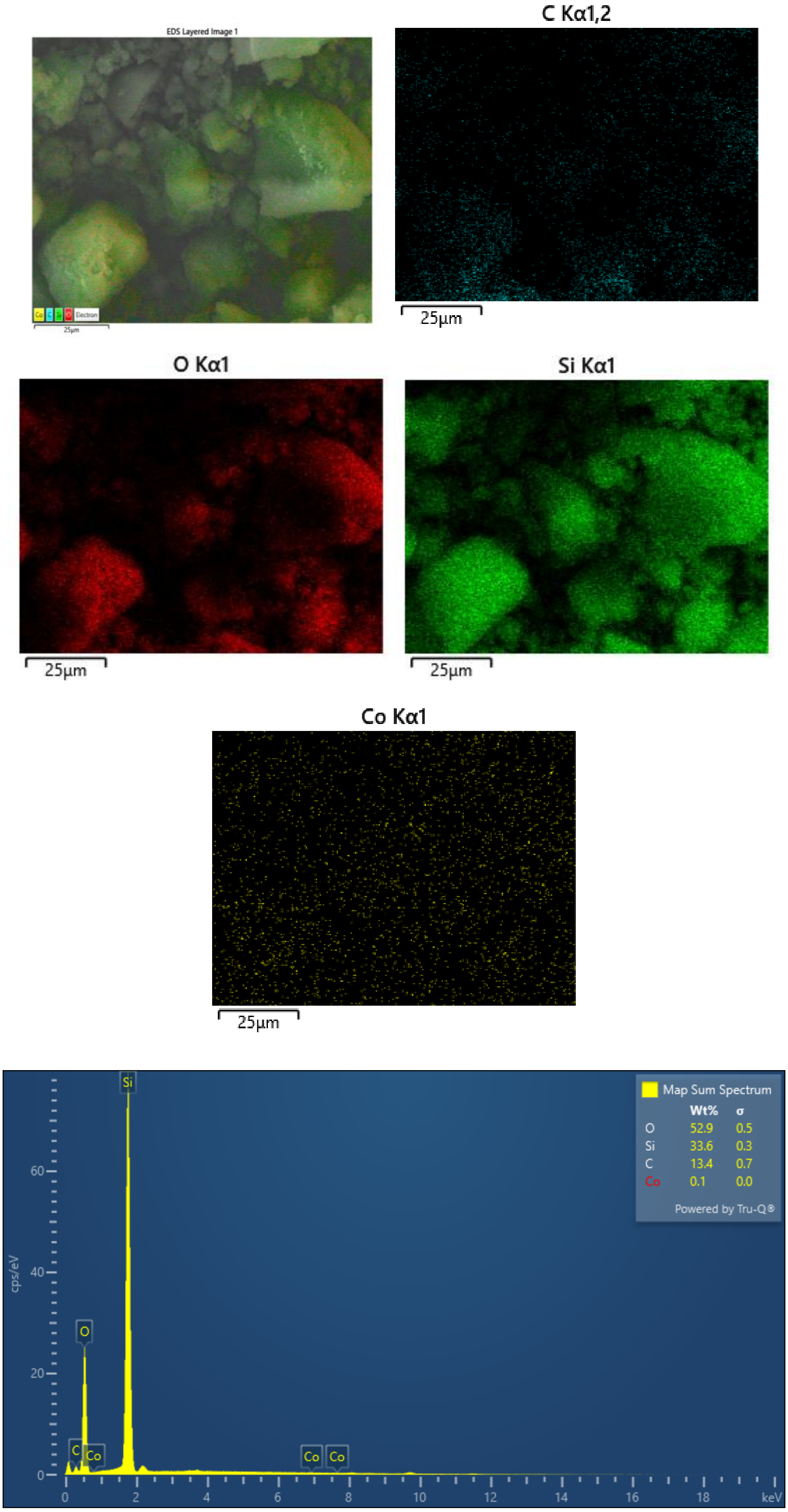

Figure S15. SEM-EDS analysis for Si-Co(3).

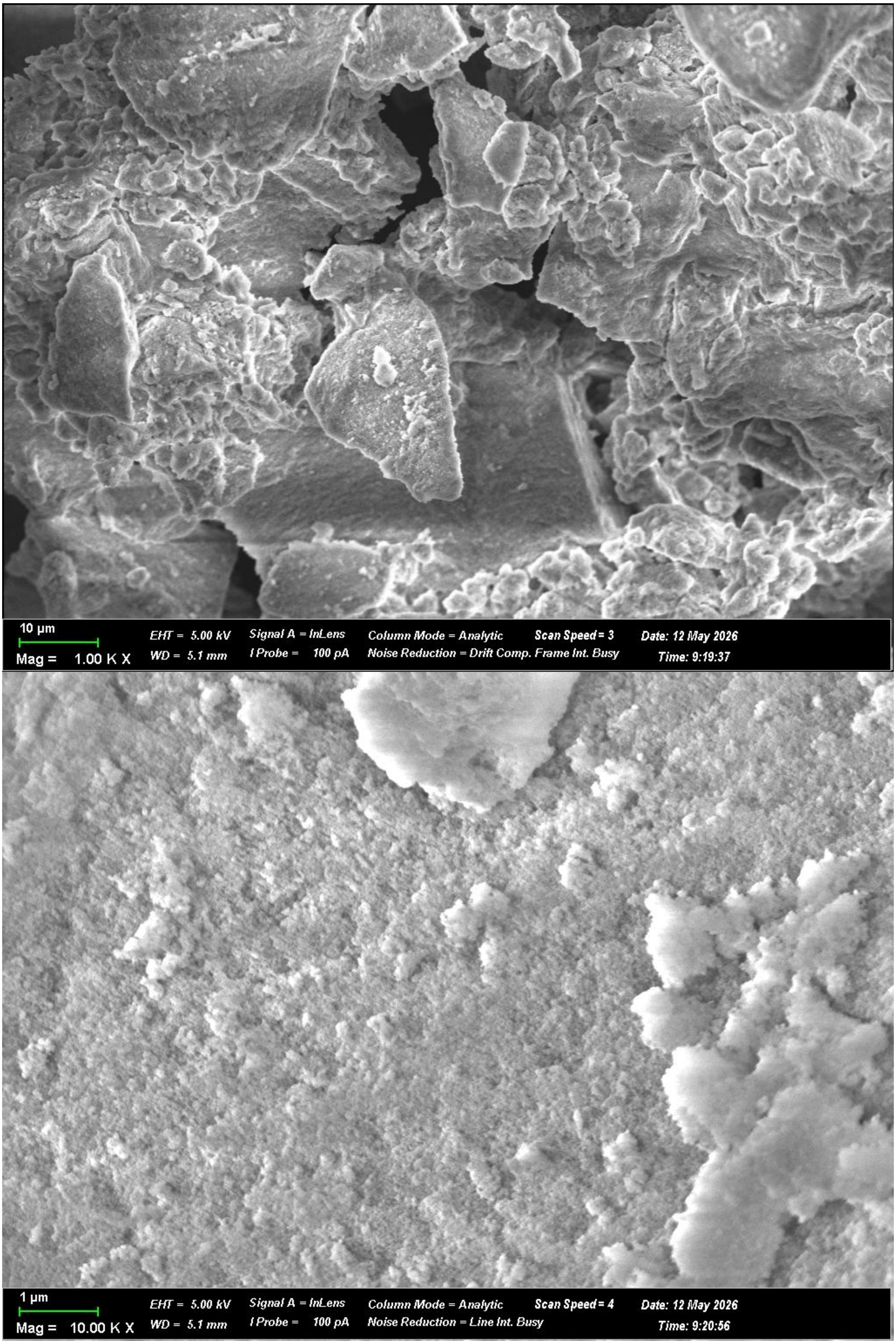

Figure S16. SEM images of TES-SiO_2.

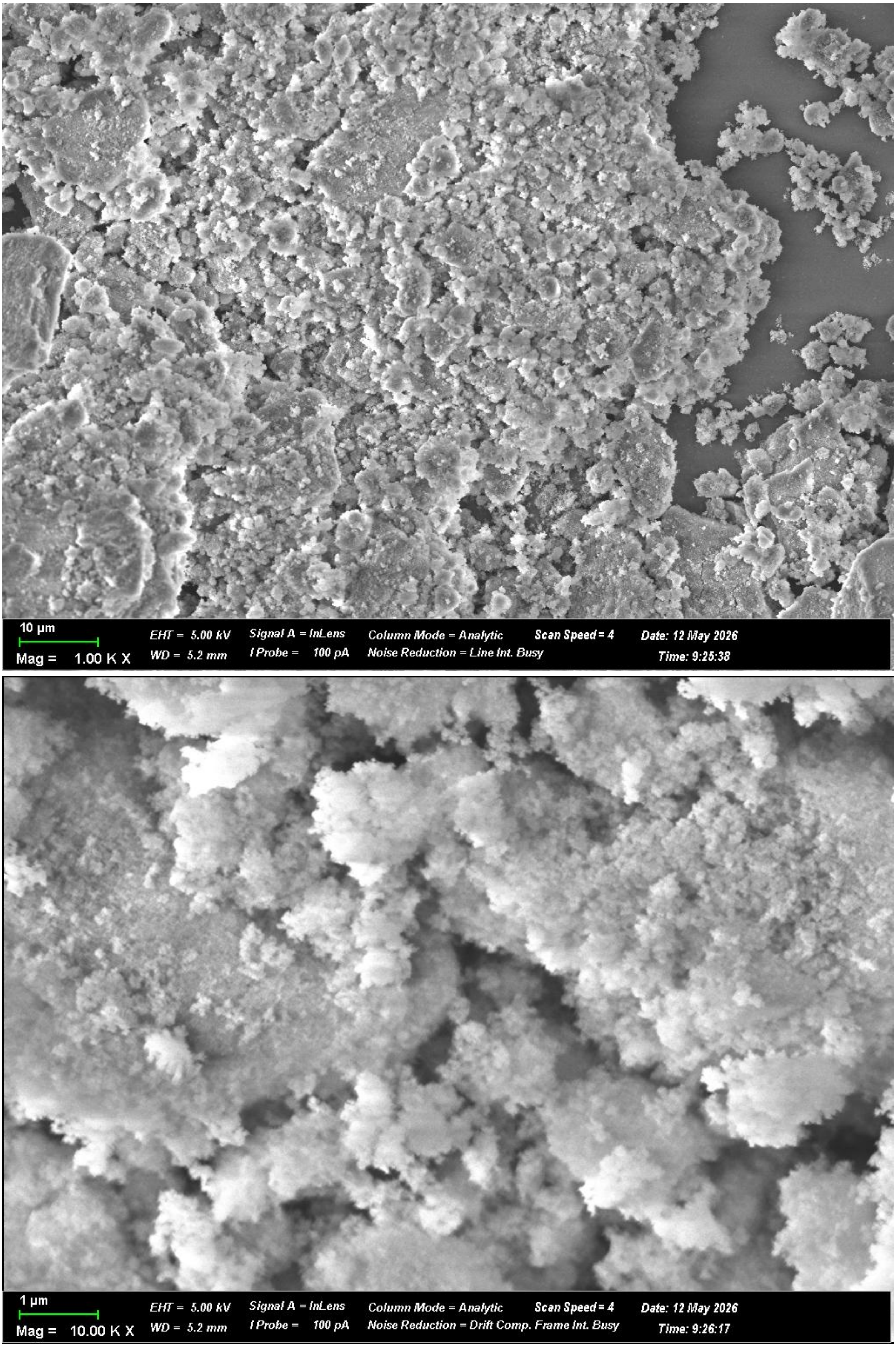

Figure S17. SEM images of Si-Co(1).

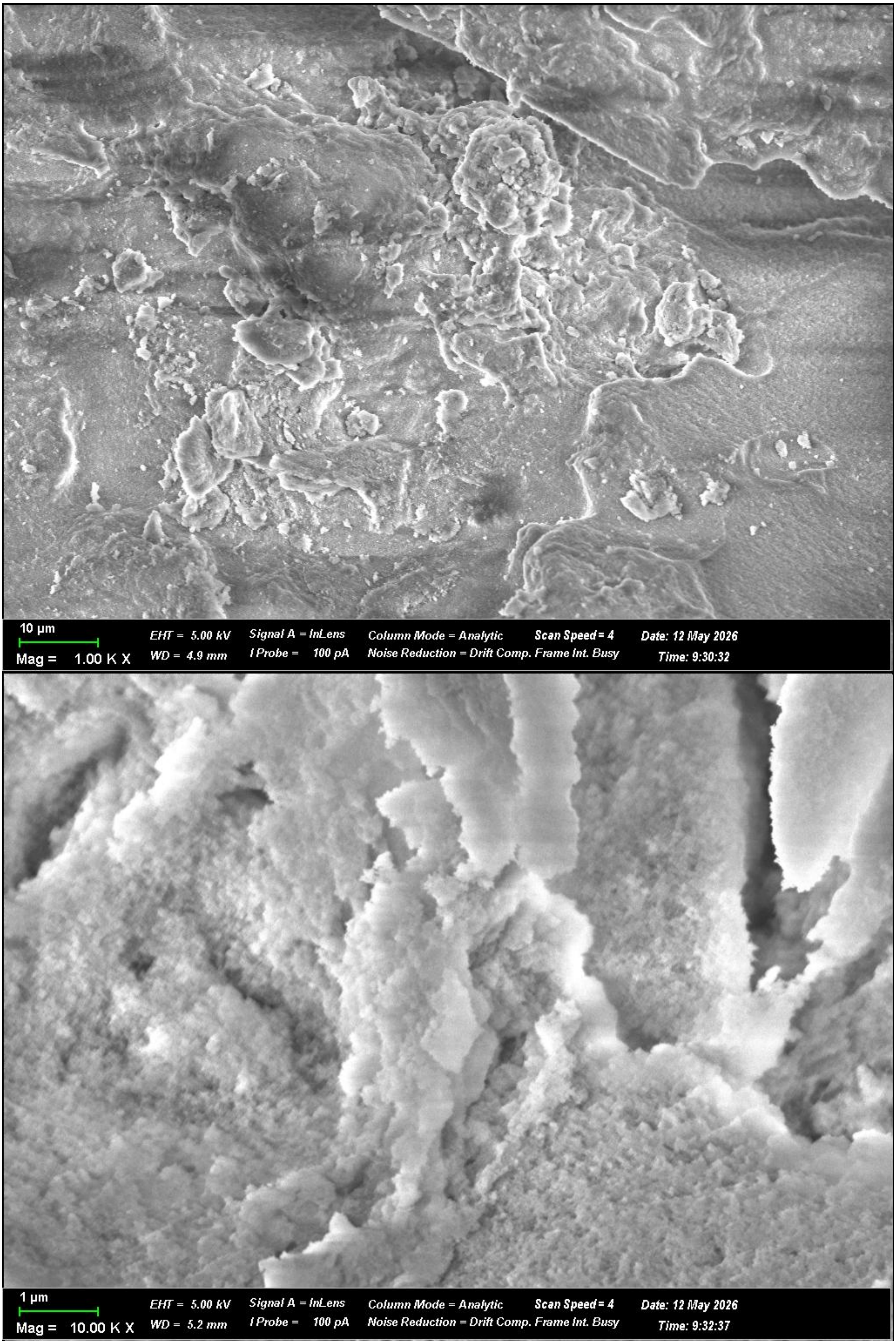

Figure S18. SEM images of Si-Co(2).

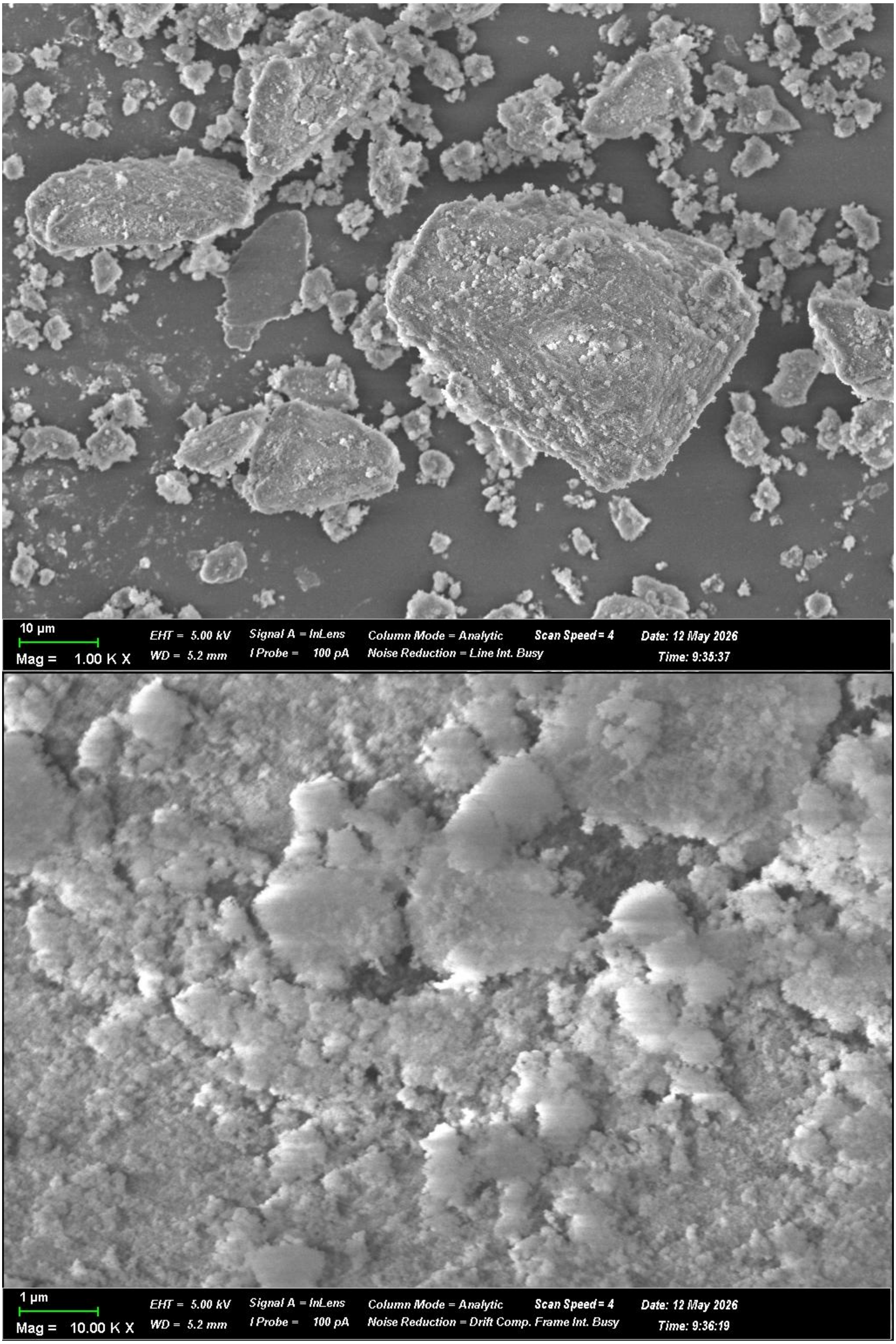

Figure S19. SEM images of Si-Co(3).

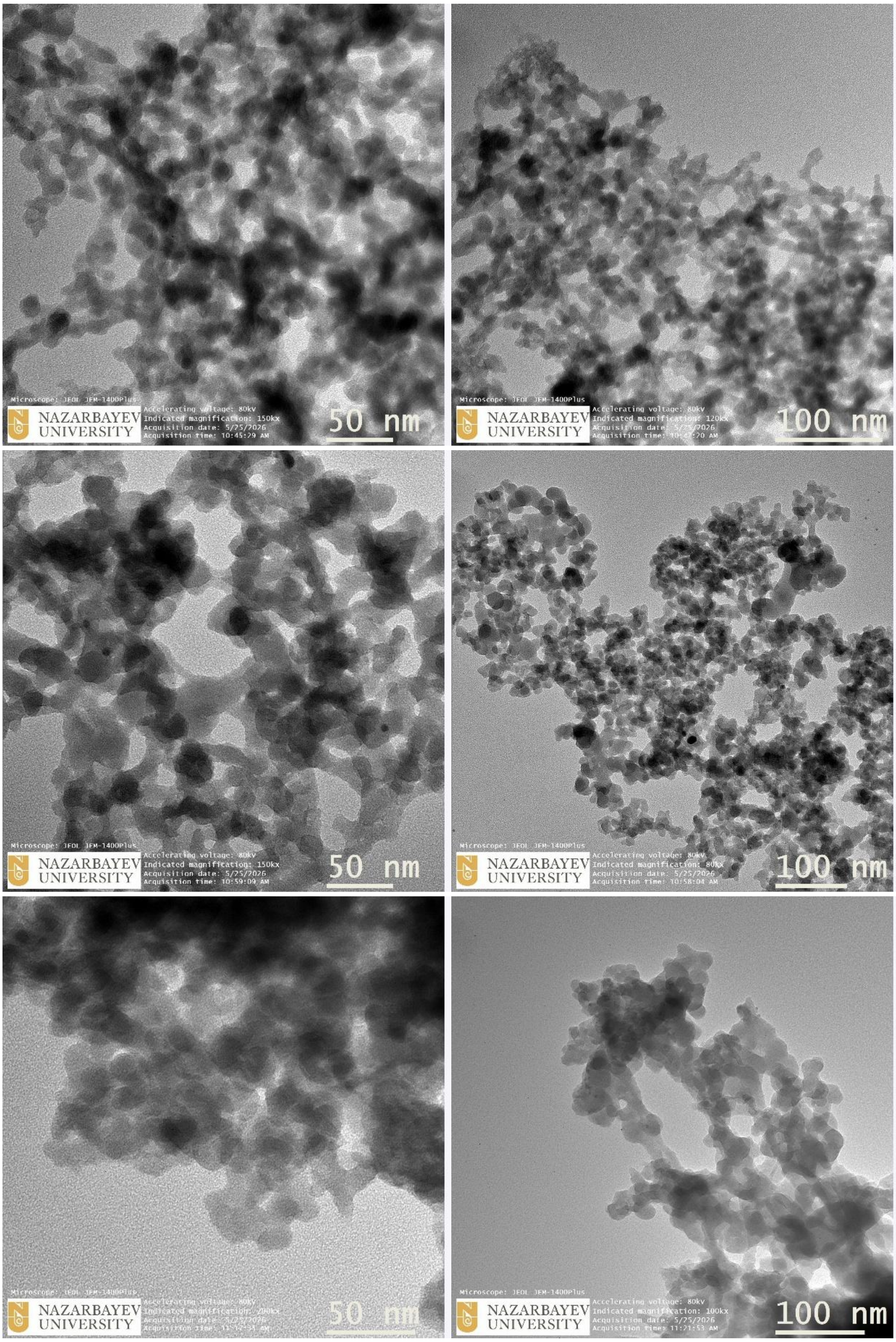

(a)

(b)

(c)

Figure S20. TEM images of fumed SiO_2 (**a**), TES-SiO_2 (**b**), and Si-Co(1) (**c**)

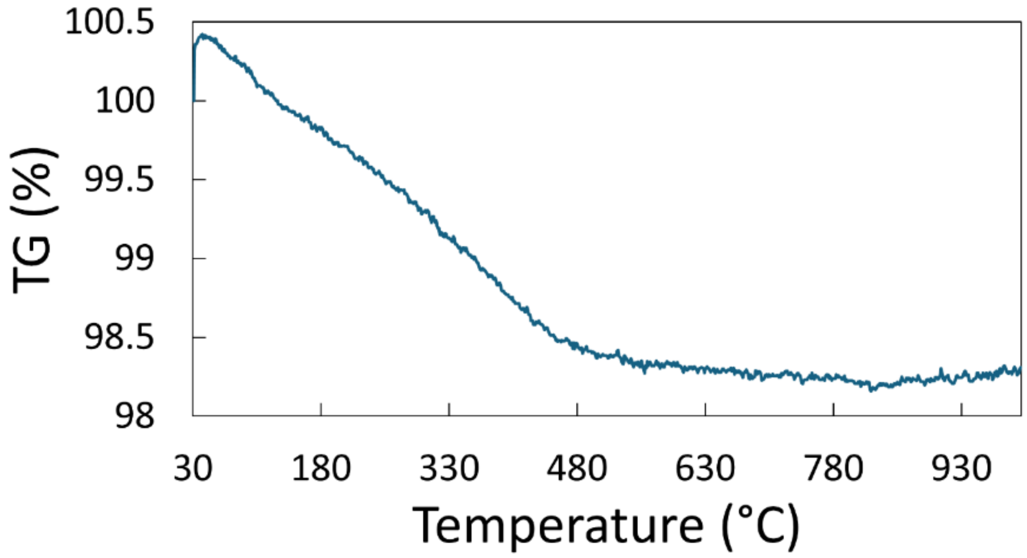

Figure S21. TGA curve for TES-SiO_2.

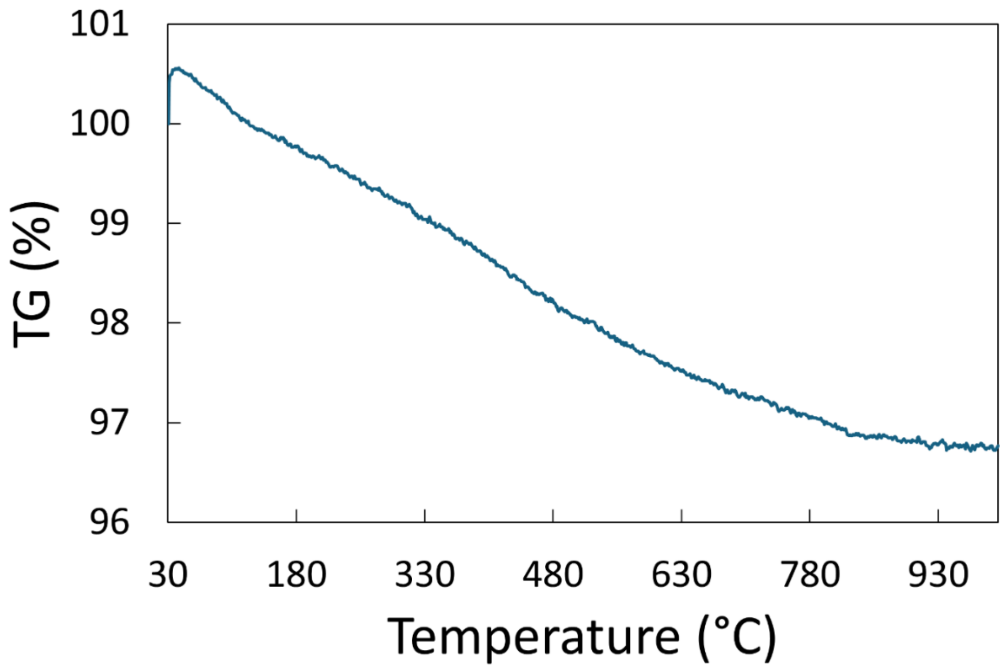

Figure S22. TGA curve for Si-Co(1).

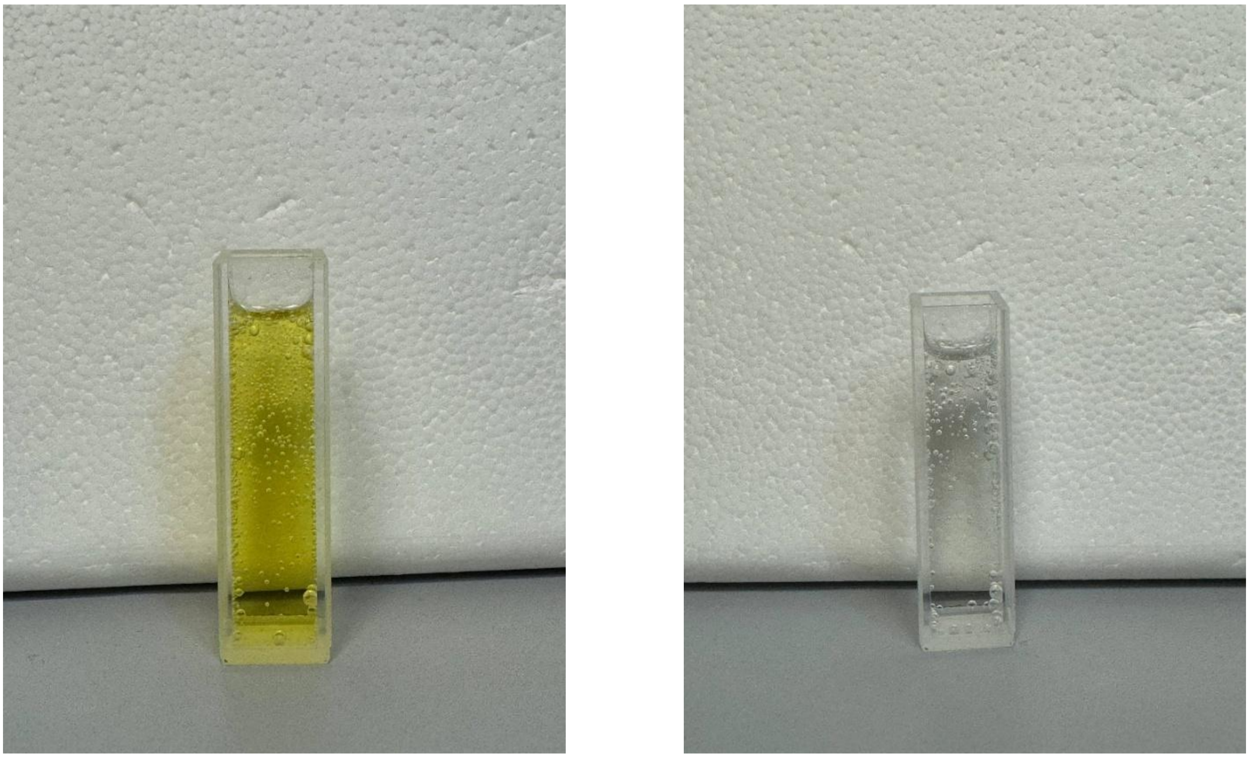

Figure S23. Reaction mixture of 2-NPh with $NaBH_4$ and cobalt (II) acetate as catalysts (30 mol% Co) at the beginning (left) and the end (after 8 min at room temperature) of the reaction (right).

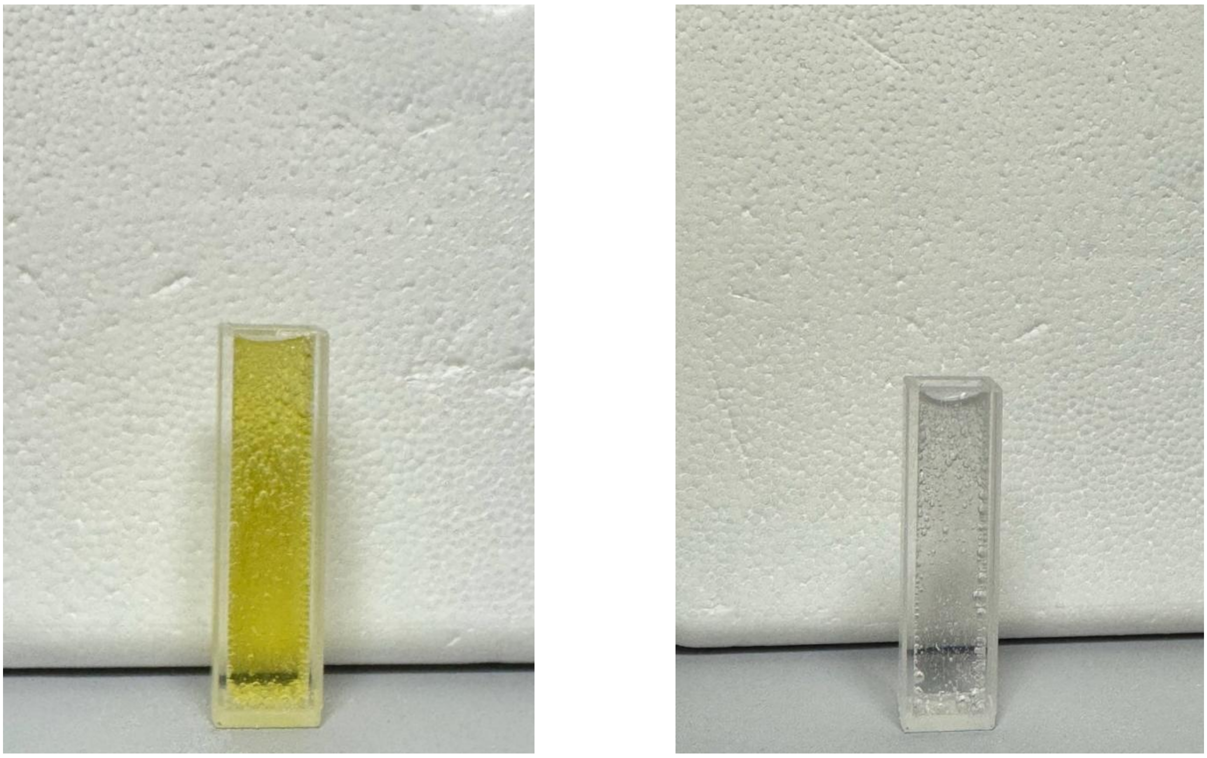

Figure S24. Reaction mixture of 2-NPh with $NaBH_4$ and Si-Co(1) as catalysts at the beginning (left) and the end (after 3 min at room temperature) of the reaction (right).

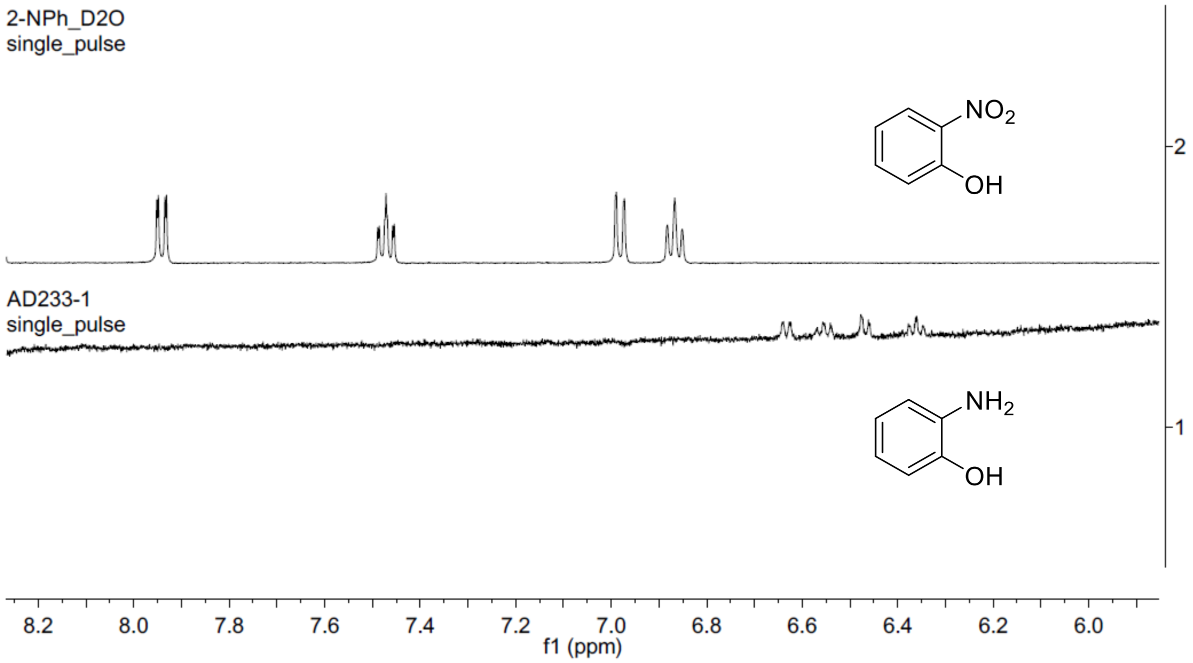

Figure S25. ^{1}H-NMR spectra (in D_2O) of 2-NPh (top) and the solid residue after Si-Co(1)-catalyzed reduction of 2-NPh with $NaBH_4$, showing complete conversion of 2-NPh and formation of 2-APh (bottom).

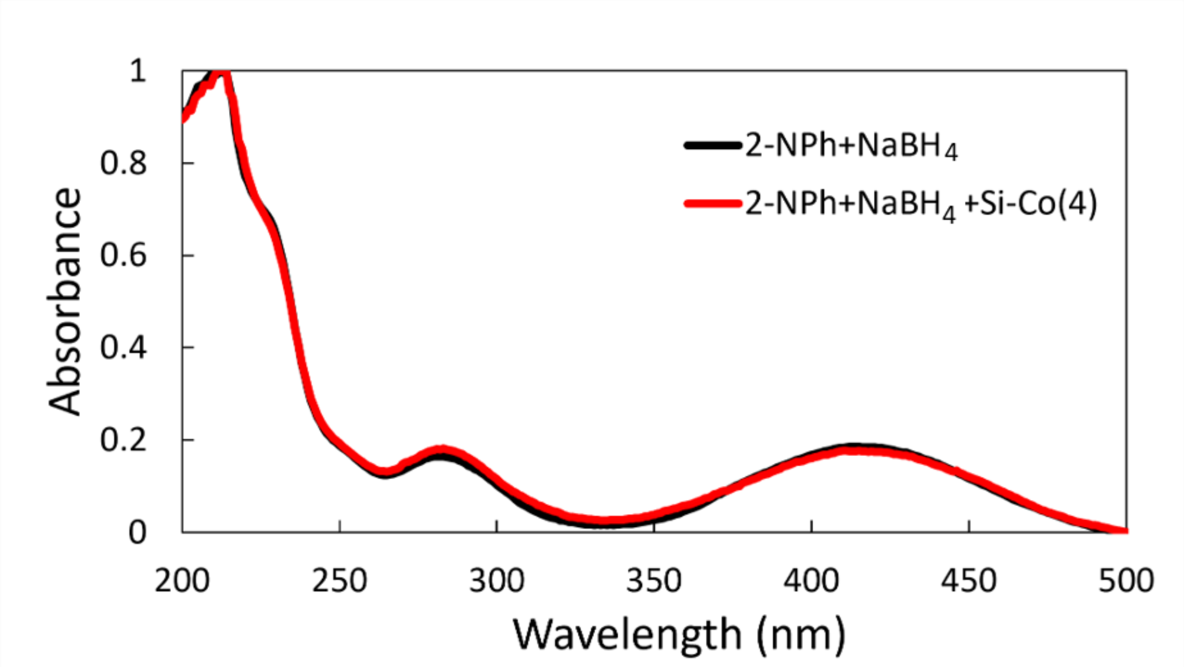

Figure S26. UV-Vis spectra taken from an aqueous solution of 2-NPh after $NaBH_4$ addition (black), and 10 min after addition of Si-Co(4) and $NaBH_4$ (red).

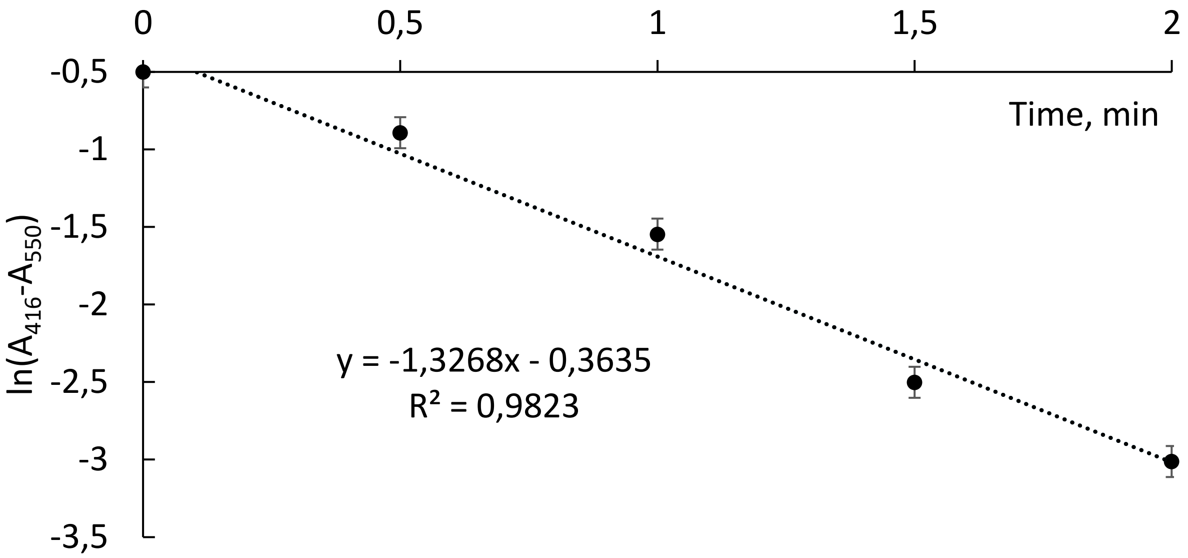

Figure S27. Reaction kinetics analysis with linear regression analysis for the reduction of 2-NPh (0.15 mM) with $NaBH_4$ (0.15 M) performed under argon atmosphere (in degassed water) using a composite prepared analogously to Si-Co(1) but under anaerobic conditions.

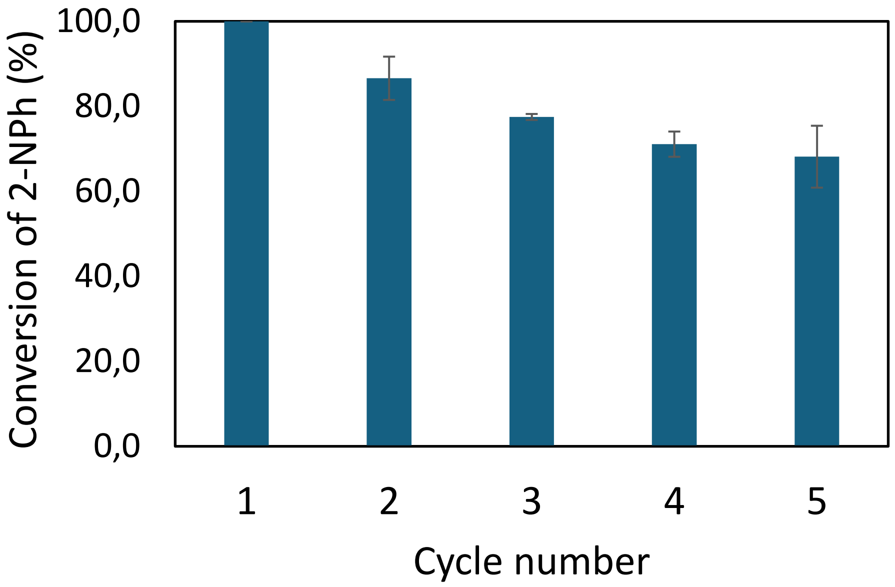

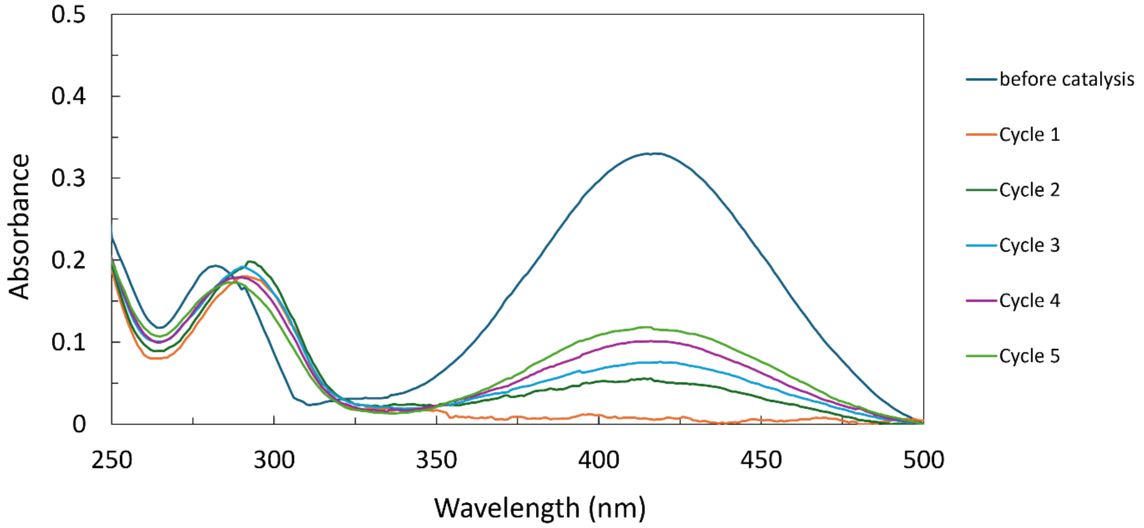

Figure S28. The reusability and recyclability of Si-Co(1) in the reduction of 2-NPh (conditions: 23.1 mg of Si-Co(1), 9 mL of H_2O, C(2-NPh) = 0.5 mmol·L^{-1}, C($NaBH_4$) = 0.25 mol·L^{-1}, 5 cycles, each cycle is 10 min at room temperature (conversions of 2-NPh: cycle 1 – 100%, cycle 2 – 87±5%, cycle 3 – 78±1%, cycle 4 – 71±3%, cycle 5 – 68±7%). Note that concentrations of 2-NPh and $NaBH_4$ did not change from the experiment described in Figure 6, the volume of each solution was increased three times along with the loading of catalyst.
